# Acute Kidney Injury Induced Lupus Exacerbation Through the Enhanced Neutrophil Extracellular Traps (and Apoptosis) in Fcgr2b Deficient Lupus Mice With Renal Ischemia Reperfusion Injury

**DOI:** 10.3389/fimmu.2021.669162

**Published:** 2021-06-24

**Authors:** Wilasinee Saisorn, Supichcha Saithong, Pornpimol Phuengmaung, Kanyarat Udompornpitak, Thansita Bhunyakarnjanarat, Peerapat Visitchanakun, Awirut Chareonsappakit, Prapaporn Pisitkun, Direkrit Chiewchengchol, Asada Leelahavanichkul

**Affiliations:** ^1^ Medical Microbiology, Interdisciplinary and International Program, Graduate School, Chulalongkorn University, Bangkok, Thailand; ^2^ Department of Microbiology, Faculty of Medicine, Chulalongkorn University, Bangkok, Thailand; ^3^ Translational Research in Inflammation and Immunology Research Unit (TRIRU), Department of Microbiology, Chulalongkorn University, Bangkok, Thailand; ^4^ Division of Allergy, Immunology, and Rheumatology, Department of Medicine, Faculty of Medicine, Ramathibodi Hospital, Mahidol University, Bangkok, Thailand

**Keywords:** Fcgr2b deficient mice, systemic lupus erythematosus, neutrophil extracellular traps, renal ischemia reperfusion injury, acute kidney injury

## Abstract

Renal ischemia is the most common cause of acute kidney injury (AKI) that might be exacerbate lupus activity through neutrophil extracellular traps (NETs) and apoptosis. Here, the renal ischemia reperfusion injury (I/R) was performed in Fc gamma receptor 2b deficient (Fcgr2b-/-) lupus mice and the *in vitro* experiments. At 24 h post-renal I/R injury, NETs in peripheral blood neutrophils and in kidneys were detected using myeloperoxidase (MPO), neutrophil elastase (NE) and citrullinated histone H3 (CitH3), as well as kidney apoptosis (activating caspase-3), which were prominent in Fcgr2b-/- mice more compared to wild-type (WT). After 120 h renal-I/R injury, renal NETs (using MPO and NE) were non-detectable, whereas glomerular immunoglobulin (Ig) deposition and serum anti-dsDNA were increased in Fcgr2b-/- mice. These results imply that renal NETs at 24 h post-renal I/R exacerbated the lupus nephritis at 120 h post-renal I/R injury in Fcgr2b-/- lupus mice. Furthermore, a Syk inhibitor attenuated NETs, that activated by phorbol myristate acetate (PMA; a NETs activator) or lipopolysaccharide (LPS; a potent inflammatory stimulator), more prominently in Fcgr2b-/- neutrophils than the WT cells as determined by dsDNA, *PAD4* and MPO. In addition, the inhibitors against Syk and PAD4 attenuated lupus characteristics (serum creatinine, proteinuria, and anti-dsDNA) in Fcgr2b-/- mice at 120 h post-renal I/R injury. In conclusion, renal I/R in Fcgr2b-/- mice induced lupus exacerbation at 120 h post-I/R injury partly because Syk-enhanced renal NETs led to apoptosis-induced anti-dsDNA, which was attenuated by a Syk inhibitor.

## Introduction

Prevalence of the dysfunction polymorphism in Fc gamma receptor 2b (Fcgr2b), which is the only one inhibitory receptor in Fc gamma receptor (FcgR) family (1–3), is usually found in Asian population. Fcgr2b is associated with systemic lupus erythematosus (SLE), a common autoimmune disease mostly associated with anti-dsDNA (4). Deficiency of the inhibitory Fcgr2b signaling causes SLE through the increased antibody production by hyper-reactive B cells (5), whereas Fcgr2b deficiency enhances the protection against malarial infection. Indeed, Fcgr2b is detectable in several immune cells (except for T cells and NK cells) (6) and the loss of Fcgr2b could induce macrophage hyper-responsiveness as demonstrated in Fcgr2b knockout (Fcgr2b-/-) macrophages (7). Additionally, Fcgr2b-/- mice develop age-dependent lupus characteristics, including asymptomatic lupus prone (less than 16 weeks old), asymptomatic lupus with anti-dsDNA (16-24 weeks old) and full-blown lupus (40 weeks old), which have been used as a representative model of lupus (8). Although the hyper-responsiveness of Fcgr2b-/- mice against several pathogen molecules, including pneumococcal antigens and lipopolysaccharide (LPS), has been demonstrated (2, 7, 8), studies on Fcgr2b-/- neutrophils are still very limited. However, lupus exacerbation is well-known for neutrophil apoptosis and neutrophil extracellular traps (NETs)-induced cell death (NETosis) (9, 10).

As such, apoptosis (cellular shrinkage, membrane blebbing and chromatin condensation) is triggered by intrinsic pathway containing the caspase 3 activation through cell damage and reactive oxygen species (ROS), and extrinsic pathway through several external death factors (11). The profound apoptosis (with an insufficient clearance) causes secondary necrosis that enhances the exposure to nuclear autoantigens (12) and accelerates anti-dsDNA production in lupus (13). Likewise, NETosis is a release of extracellular DNA networks by neutrophils in either infectious or non-infectious condition (14) through peptidylarginine deiminase 4 (PAD4)-induced citrullinated histone H3 (CitH3) (15). Extracellular DNA networks from NETosis, both of NADPH oxidase 2 (NOX2)-dependent and NOX2-independent pathways, enhance exposure of nuclear contents (16), including dsDNA that is normally contained in nuclei (17), and increased anti-dsDNA which is a specific auto-antibody in lupus (18, 19). With profound cell death, the free dsDNA is recognized as a damage associated molecular patterns (DAMPs) by innate immune cells (17) and processed by adaptive immune cells supporting the lupus exacerbation from inflammatory responses (20, 21). Not only cell death of the immune cells, but also the dying process of other cell types, exacerbate lupus as the avoidance of ultraviolet light, that induces keratinocyte cell death (22–24), is a current mandatory recommendation for lupus (17). Due to the abundance of neutrophils, cell death of neutrophils in the host with lupus-prone condition (loss of tolerance) (25), but not the normal host, might profoundly enhance the exposure of dsDNA that increases anti-dsDNA production. Besides, the susceptibility to program cell death of Fcgr2b-/- neutrophils might be higher than WT cells that have been triggered the lupus activity.

In parallel, acute kidney injury (AKI) is the common health care problem worldwide, which is mainly caused by ischemia (26) that was often represented by renal ischemia reperfusion injury (I/R) animal model (27, 28). As such, renal ischemia induces accumulation of immune cells, including neutrophils, as a response to the injury (29–31) and the injury also causes apoptosis (and necrosis) in renal parenchymal cells and neutrophils (32, 33). Consequently, the ischemic injury directly induces program cell deaths of neutrophils, including NETosis, in several organs after renal injury (34). After the renal ischemia, cell apoptosis and NETosis might be exacerbated the lupus disease activity due to the lupus exacerbation from the program cell deaths (35). Because of the prominent responses against several stimulators of the Fcgr2b-/- mice (2, 7, 8), apoptosis and NETosis derived from renal I/R might be more profound than the responses in the normal mice. TLR-4 is the significant pathway that induces both apoptosis and NETosis (36), while spleen tyrosine kinase (Syk) is the shared downstream signaling pathway of TLR-4 and FcgR (20, 21, 37, 38). Therefore, the Syk inhibitor may be an interesting drug to manipulate cell death in renal-I/R mediated cell death in lupus (39). Indeed, the Syk inhibitor (fostamatinib, previously known as R788) is a US FDA (the United States Food and Drug Administration) approved drug for autoimmune diseases (40–43). Here, we reported the *in vitro* and *in vivo* experiments to determine the effect of renal-I/R on lupus and evaluated the Syk inhibitor on Fcgr2b-/- lupus mice.

## Materials and Methods

### Animals

The animal study protocol was approved from the Institutional Animal Care and Use Committee of the Faculty of Medicine, Chulalongkorn University, Bangkok, Thailand, under approval number 009/2564, following the animal care and use protocol of the National Institutes of Health (NIH), USA. Fcgr2b-/- mice on a C57BL/6 background were provided by Dr. Silvia Bolland (NIAID, NIH, Maryland, USA). The wild-type (WT) mice were purchased from the Nomura Siam International (Pathumwan, Bangkok, Thailand). Due to the age-dependent lupus characteristic (44, 45), 8-week-old Fcgr2b-/- female mice represented asymptomatic lupus prone mouse model and age-matched female WT mice were used in all experiments.

### Renal Ischemia Reperfusion Injury Model and NETs Inhibitors

Renal I/R was performed following a previous publication (27). In brief, bilateral renal arteries were clamped for 35 min through the abdominal incision under ketamine anesthesia on a 37°C heated operation table. In sham surgery, renal arteries were only identified before closing the abdominal wall. Tramadol, 20 mg/kg diluted in 0.5 mL normal saline (NSS) was administered subcutaneously after surgery and at 24 h post-I/R. Mice were sacrificed in several time-points after I/R under isoflurane anesthesia for sample collection. Serum was kept at −80°C until analysis and organs were processed in 10% formalin or Tissue-Tek O.C.T Compound (Sakura Finetek, CA, USA) for histological analysis or snap frozen and stored separately at -80°C. The Syk inhibitor was used for testing the anti-inflammatory effect (42) and a possible association between activating-FcgRs and Syk signaling (46). As such, a Syk inhibitor (R788 disodium, Selleckchem, Houston, USA) in 0.1 M citrate buffer (pH 6.8) at 25 mg/kg/dose was orally administered daily for 2 days, at 6 h prior to surgery, and at 6 h after renal-I/R. In parallel, a Cl-amidine (PAD4 inhibitor; Sigma-Aldrich, St. Louis, MO, USA) was dissolved in dimethyl sulfoxide (DMSO; Sigma-Aldrich). The stock solution was dissolved in normal saline at 10 mg/kg/dose and injected intraperitoneal 3 h prior to renal-I/R at once daily after following a previous publication (47) to test the influence of NETs in lupus model with renal-I/R. Mice were sacrificed with sample collection under isoflurane anesthesia.

### Blood and Urine Analysis

Parameters of lupus characteristics, serum cytokines, liver injury, and NETosis were evaluated from blood samples. Lupus characteristics including serum creatinine (QuantiChrom Creatinine-Assay, DICT-500, BioAssay, Hayward, CA, USA), serum anti-dsDNA, and proteinuria were determined. Symptomatic lupus was defined as increased serum anti-dsDNA, high serum creatinine, and increased proteinuria when compared with the WT mice. The anti-dsDNA was evaluated using a protocol with coated-calf thymus DNA (Invitrogen, Carlsbad, CA, USA) with a minor modification (48). Briefly, each analyzed plate was coated with calf thymus DNA (for dsDNA) and ssDNA that was prepared using the thermal denaturation of calf thymus DNA (49). Briefly, the plates were incubated overnight at 4°C, filled with blocking solution at room temperature, and washed with 1X TBS 0.05% Tween 20, respectively. Subsequently, mouse serum samples were added into the plates and incubated overnight at 4°C. Then, HRP-conjugated goat anti-mouse antibodies and TMB peroxidase substrate (TMB Substrate Set; BioLegend, San Diego, CA, USA) were added to analyze the plates, and 2 N H_2_SO_4_ was added to stop the reaction. The measurement at a wavelength of 450 nm was determined using a Varioskan Flash spectrophotometer (Thermo Scientific, Waltham, MA, USA). The reported anti-dsDNA was represented from the values of calf-DNA coated plates subtracted by ssDNA-coated plates.

Proteinuria was calculated using spot urine protein creatinine index (UPCI) with the equation; UPCI = urine protein (mg)/urine creatinine (mg/dL). Urine protein and creatinine were measured by Bradford Bio-Rad Protein Assay (Bio-Rad, Hercules, CA, USA) and QuantiChrom Creatinine-Assay (DICT-500) (BioAssay), respectively. Serum pro-inflammatory cytokines (TNF-α and IL-6) and IL-1β, a NETs associated cytokine (50), were measured using enzyme-linked immunosorbent assay (ELISA) (Invitrogen). Liver injury was determined by alanine transaminase (ALT) using EnzyChrom ALT assay (EALT-100, BioAssay). For the evaluation of NETs in peripheral blood neutrophils, neutrophils were isolated using Polymorphprep (Alere Technologies AS, Norway) according to the manufacturer’s instructions (51, 52), and hypotonic lysis buffer was used for red blood cell de-contamination. Blood neutrophils were resuspended in RPMI (Roswell Park Memorial Institute media)-1640 media and the purity was assessed by Wright’s stains. The samples with >95% neutrophils were further used to determine the NETs formation as mentioned in the section of *in vitro* experiments. Additionally, NETs formation in serum was also determined using Quant-iT™ PicoGreen dsDNA Assay Kit (Thermo Scientific), and serum MPO was measured by ELISA (Sigma Aldrich, St. Louis, MO, USA).

### Polymerase Chain Reaction

Several molecules associated with inflammation (cytokines), neutrophil extracellular traps (NETs), and the possible downstream signals from mouse organs and the cell culture were evaluated by real time polymerase chain reaction (RT-PCR). Gene expression of several molecules, including inflammatory cytokines; *TNF-α, IL-6*, and *IL-10*, genes of NETs formation; peptidyl arginine deiminase 4 (*PAD4*) and *IL-1β*, genes of the downstream signals; spleen tyrosine kinase (*Syk*) and nuclear factor kappa B (*NFκB*), were evaluated. Total RNA was prepared from the samples with an RNA-easy mini kit (Qiagen, Hilden, Germany) and was quantified by Nanodrop 100 Spectrophotometer (Thermo Scientific) before the determination of gene expression. Total RNA reverse transcription was processed with a High-Capacity cDNA Reverse Transcription (Thermo Scientific). Samples were performed using SYBR Green PCR Master Mix for quantitative RT-PCR with QuantStudio6 Flex Real-time PCR System (Thermo Scientific), respectively. The results were demonstrated in terms of relative quantitation of the comparative threshold (delta-delta Ct) method (2-ΔΔCt) as normalized by *β-actin* (an endogenous housekeeping gene). The list of primers is shown in **Supplementary Table 1**.

### Histological Analysis and Immunofluorescent Imaging

The semi-quantitative evaluation of renal and lung histology on paraffin-embedded slides was performed after 10% neutral buffered formalin fixation, followed by Hematoxylin and Eosin (H&E) staining at 200× magnification in 10 randomly selected fields for each animal (53–55). Renal injury was defined as tubular epithelial swelling, loss of brush border, vacuolar degeneration, necrotic tubules, cast formation, and desquamation using the scoring method as follows: 0, area of damage <5%; 1, area of damage 5%–10%; 2, area of damage 10%–25%; 3, area of damage 25%–50%; and 4, area of damage >50%. Lung injury was determined by alveolar hemorrhage, alveolar congestion, neutrophil infiltration, and alveolar wall thickness with the following score: 0, no injury in the observed field;1, injury up to 25%; 2, injury up to 50%; 3, injury up to 75%; and 4, injury in the entire field.

In parallel, immunofluorescent histological analysis was performed following previous publications (8, 13, 56). In brief, the internal organs were prepared in Tissue-Tek O.C.T Compound (Sakura Finetek, CA, USA) and three sections of each organ were stained as follows; i) NETs were detected with antibody against neutrophil elastase (NE; ab68672), myeloperoxidase (MPO; ab25989), and citrullinated histone H3 (citrulline R2 + R8 + R17; ab5103) (Abcam, Cambridge, MA, USA) with DAPI (4′,6-diamidino-2-phenylindole), a blue-fluorescent DNA stain (Sigma Aldrich), ii) cell apoptosis was visualized by anti-Cleaved Caspase 3 (Asp175, 9661S) (Cell Signaling Technology, Boston, MA, USA) with the secondary antibodies; goat anti-rabbit IgG (ab150077) (green color), goat anti-mouse IgG (ab150115), and donkey anti-rabbit IgG (ab150075) (red color) and iii) immunoglobulin deposition was performed using goat anti-mouse IgG (green color) (ab150113; Abcam, Cambridge, MA, USA). The images were analyzed with ZEISS LSM 800 (Carl Zeiss, Germany).

### Flow Cytometry Analysis of Spleen

Spleen lymphocytes were explored to compared between Fcgr2b-/- and WT mice after 120 h post renal-I/R (or sham), were following a previous published protocol (41). In short, spleens were dispersed through a cell strainer to generate a single-cell suspension in supplemented RPMI-1640 prior to centrifuged at 300 *× g* for 5 min at 4°C. Red blood cells were eliminated using an osmotic agent (ACK buffer; NH4Cl, KHCO3, and EDTA). Then, the cells were resuspended in staining buffer (0.5% bovine serum albumin and 10% fetal bovine serum in PBS), and were stained with fluorochrome-conjugated antibodies against different mouse immune cells, including anti-B220 (leukocyte common antigen) together with anti-CD138 (plasma cells), anti-major histocompatibility complex (MHC) class II (active immune cells), CXCR5 (follicular B cells), and anti-CD19 along with anti-GL-7 (germinal center B cells) (BioLegend, San Diego, CA, USA). All stained cells were examined by flow cytometry using BD LSR-II (BD Biosciences) and the data were analyzed by FlowJo software (Tree Star Inc., Ashland, OR, USA).

### The *In Vitro* Experiments on Neutrophils

The apoptosis and NETosis susceptibility between Fcgr2b-/- and WT neutrophils, and the attenuation by inhibitors were tested using the *in vitro* experiment. Accordingly, neutrophils were derived from peritoneum using a published protocol (57). Briefly, 1 mL of 3% thioglycolate was intraperitoneal administered in 8-week-old mice. At 3 h after administration, mice were sacrificed and peritoneal cavity was thoroughly collected and washed with ice-cold phosphate buffer solution (PBS) before centrifugation at 1,800 *× g*, 4°C for 5 min to separate the cells and evaluated by Wright’s-stains (55). Only the preparation with more than 90% neutrophils was further used for the experiments. Then, neutrophils at 2 × 10^5^ cells/well in 24-well-plates containing RPMI media were incubated with phorbol myristate acetate (PMA), a NETs stimulator, (Sigma-Aldrich) at a final concentration of 25 ng/mL or lipopolysaccharide (LPS) (Escherichia coli 026: B6; Sigma-Aldrich) at 100 ng/mL under 5% CO_2_ at 37°C for 4 h. Supernatants were used for cytokine measurement by ELISA (Invitrogen) and were quantified for dsDNA using PicoGreen assay kit (Invitrogen, Canada) following the manufacturer’s protocol. Nuclei morphology has been identified with DAPI nuclear staining (58), which was presented by the percentage of cells with NETs formation, and *PAD4* expression was determined using RT-PCR as the previously mentioned. Additionally, neutrophils were suspended in PBS at a concentration of 5 × 10^5^ cells/mL, stained for apoptosis/necrosis by annexin V-FITC and propidium iodide (PI) (5 µL/well) (BD Biosciences), respectively. Then, the samples were washed with FACS flow buffer, PBS supplemented with 1% (v/v) FBS, and 0.05% NaN_3_ and processed by the BD LSR II Flow Cytometry (BD Biosciences) using the FlowJo software (Tree Star Inc.). Reactive oxygen species (ROS) was determined by DHE (Dihydroethidium) assay (ab236206; Abcam, Cambridge, MA, USA), according to the manufacturer’s instructions. In addition, the active metabolites Syk inhibitor (R406; Selleckchem) at a final concentration of 5 µg/mL or nuclear factor kappa B (NFκB) inhibitor (BAY11-7082; Sigma–Aldrich) at a final concentration of 2 µg/mL were used in the experiments. RPMI 1640 supplemented with 10% FBS was used as the control.

### Western Blot Analysis

Western blot analysis was performed as previously described (59) to determine the abundance of Syk and NFκB in activated neutrophils. In brief, cell lysate (in 1X SDS lysis buffer) was supplemented with the inhibitors against protease and phosphatase enzymes (Thermo-Scientific) and incubated on ice for 30 min before centrifugation at 10,000 rpm at 4°C. Protein quantification was performed by BCA assay (Pierce BCA Protein Assay) before administration in the 10% SDS-PAGE (Sodium Dodecyl Sulfate-Polyacrylamide Gel Electrophoresis) and transferred onto a nitrocellulose membrane, respectively. Then, the membranes were blocked with Intercept blocking buffer (Lincoln, NE, USA) and incubated with primary antibodies against Syk (D3Z1E), phospho-Zap-70 (Tyr319)/Syk (Tyr352) (65E4) (Cell Signaling Technology, Boston, MA, USA), NFκB p65 (NFκB; D14E12) and phospho-NFκB p-65 (p-NFκB (Ser536) (93H1); Cell Signaling Technology), and Beta-actin (β-actin, a house-keeping protein) (D6A8; Cell Signaling Technology). Subsequently, the secondary antibody is anti-rabbit IgG (DyLight 680 conjugate) was used and visualized by Odyssey CLx imaging system (Lincoln, NE, USA). The target bands were determined using Image Studio Lite version 5.2.

### Statistical Analysis

Statistical differences among groups were examined using the unpaired Student’s t-test or one-way analysis of variance (ANOVA) with Tukey’s comparison test for the analysis of experiments with two groups or more than two groups, respectively, all of which are presented as the mean ± standard error (SE). Statistical comparisons of data were conducted by paired Student’s t-test in the experiment condition of before and after treatment. SPSS 11.5 software (SPSS, Chicago, IL, USA) was used for all statistical analysis.

## Results

Although the renal I/R induced the renal excretory dysfunction (serum creatinine) at 24 h post-renal I/R of Fcgr2b-/- mice was similar to those of wild type (WT) mice, there was higher NETs and apoptosis in glomeruli which led to lupus nephritis possibly through Syk signaling after 120 h post-I/R.

### Similar Organ Injury but More Prominent Neutrophil Extracellular Traps (NETs) and Glomerular Apoptosis at 24 h Post-Renal I/R in Fcgr2b-/- Lupus Mice Compared With WT

**Figure 1:**
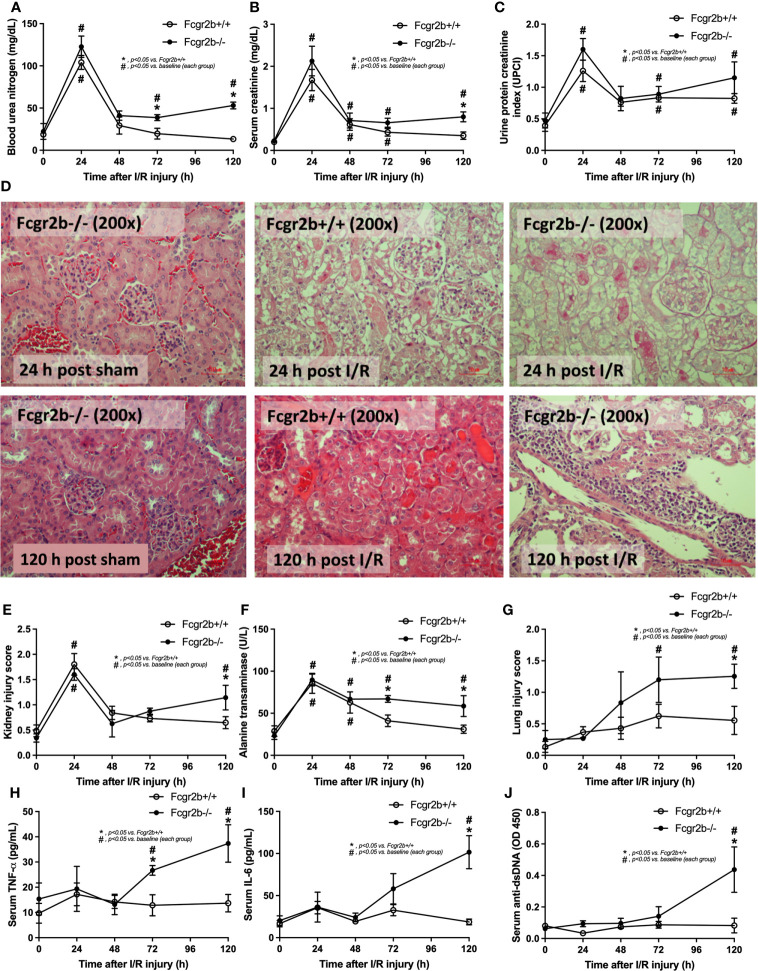
Renal ischemia reperfusion injury (I/R) at 24 h induced lupus disease exacerbation at 120 h. The time-point characteristics of Fcgr2b-/- or wild-type (Fcgr2b+/+) mice after I/R as determined by i) renal injury; blood urea nitrogen (BUN), serum creatinine, and proteinuria (protein creatinine index; UPCI) along with the histological score of the representative images in Hematoxylin and Eosin (H&E) staining (original magnification 200x) **(A–E)**, ii) liver injury (serum alanine transaminase) **(F)**, iii) lung injury score **(G)**, and iv) the parameters in serum; serum pro-inflammatory cytokines (TNF-α and IL-6) **(H, I)** and serum anti-dsDNA, a major auto-antibody in lupus **(J)** are demonstrated (n = 6–7/time-point). Notably, the renal histological pictures at 24 h post sham of Fcgr2b+/+ mice and at 120 h post sham (both mouse strains) are not demonstrated due to the similarity to Fcgr2b-/- mice at 24 h post sham (normal histology).

**Figure 2:**
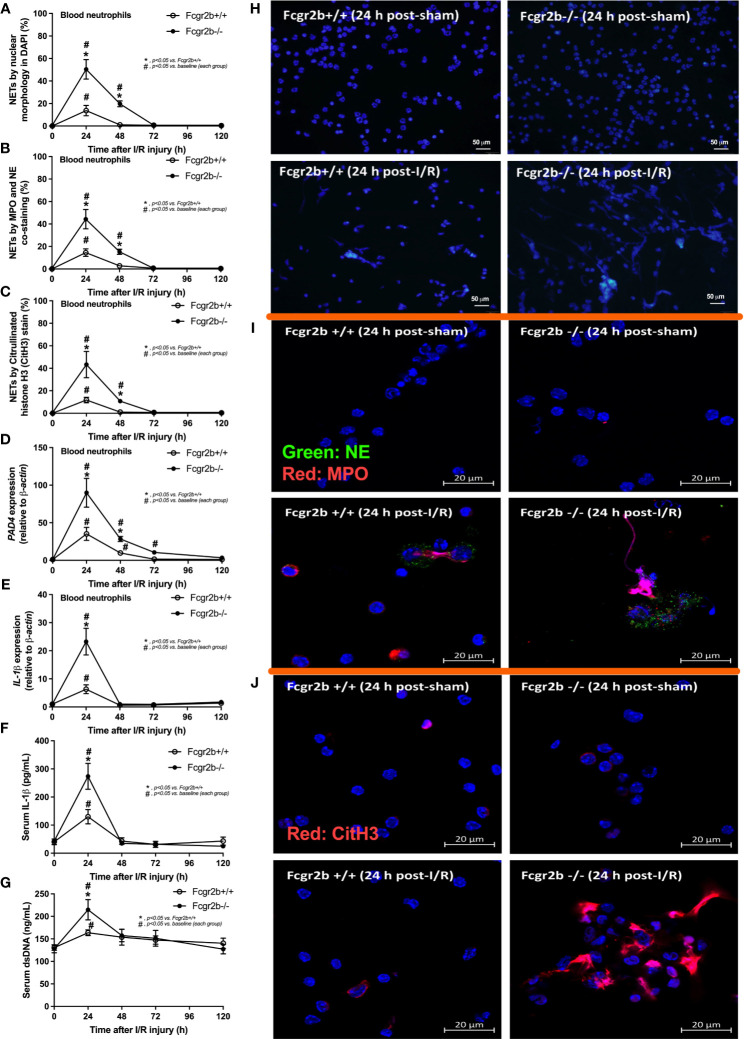
Renal ischemia reperfusion injury (I/R) at 24 h induced more prominent neutrophil extracellular traps (NETs) in blood of lupus prone mice. Characteristics of NETs in peripheral blood neutrophils from Fcgr2b-/- or wild-type (Fcgr2b+/+) mice after renal ischemia reperfusion injury (I/R) as determined by the nuclear morphology by 4’,6-diamidino-2-phenylindole (DAPI) staining (blue color) **(A)**, co-staining of myeloperoxidases (MPO) and neutrophil elastase (NE) **(B)**, citrullinated histone H3 (CitH3) staining **(C)** with the representative pictures from peripheral blood neutrophils at 24 h post-I/R (or sham) from both mouse strains with DAPI staining (original magnification 200x) **(D)**, and immunofluorescence of MPO/NE and CitH3 (original magnification 630x) **(E, F)** are demonstrated. Additionally, the gene expression of *PAD4* and *IL-1β* **(G, H)** together with serum IL-1β **(I)** and serum ds-DNA **(J)** are also demonstrated (n = 6–7/time-point).

**Figure 3:**
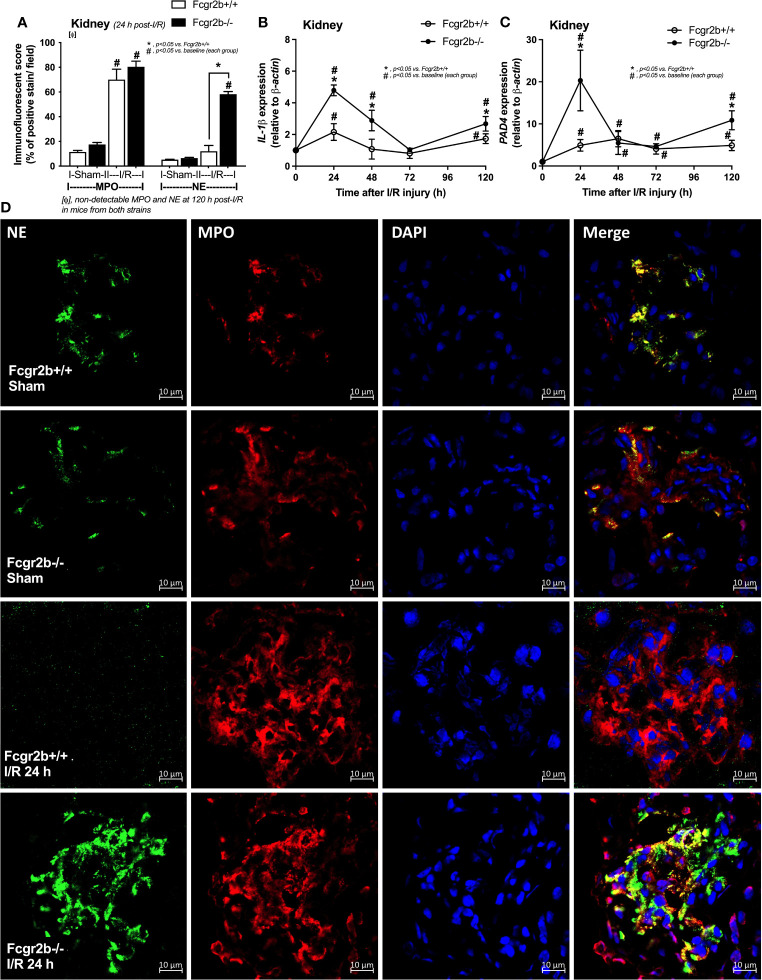
Renal ischemia reperfusion injury (I/R) at 24 h induced more prominent neutrophil extracellular traps (NETs) in renal glomeruli of lupus prone mice. Characteristics of NETs in kidneys from Fcgr2b-/- or wild-type (Fcgr2b+/+) mice after renal ischemia reperfusion injury (I/R) as determined using co-detection of myeloperoxidases (MPO) and neutrophil elastase (NE) at 24 h post-I/R **(A)** (n = 5–8/group) and gene expression in the time-point of *IL-1β* and *PAD4* **(B, C)** (n = 6–7/time-point) with the representative immunofluorescent pictures of NE (green), MPO (red) and 4’,6-diamidino-2-phenylindole (DAPI, blue nuclear staining) at 24 h post-I/R (or sham) (original magnification 630x) **(D)** are demonstrated.

**Figure 4:**
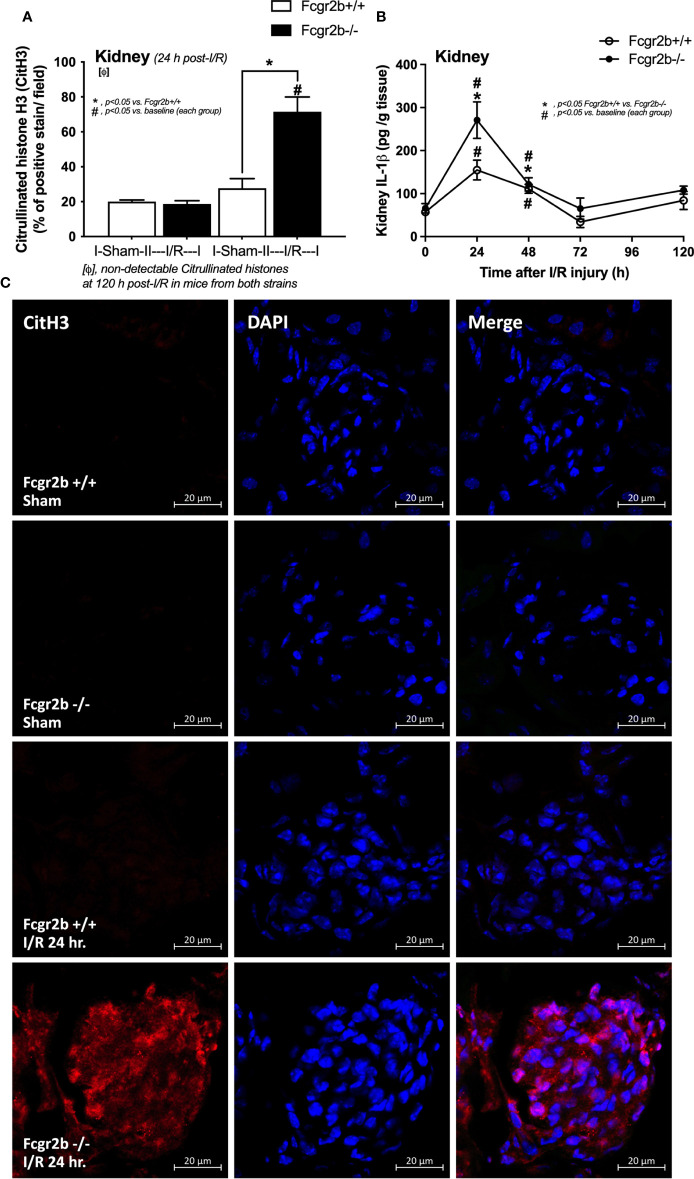
Renal ischemia reperfusion injury (I/R) at 24 h induced more prominent neutrophil extracellular traps (NETs) in renal glomeruli of lupus prone mice. Characteristics of NETs in kidneys from Fcgr2b-/- mice or wild-type (Fcgr2b+/+) mice after renal ischemia reperfusion injury (I/R) as determined by immunofluorescent staining of citrullinated histone H3 (CitH3) at 24 h post-I/R **(A)** (n = 5–6/group) and IL-1β from renal tissue **(B)** (n = 5–6/time-point) with the representative immunofluorescent pictures of CitH3 (red) and 4’,6-diamidino-2-phenylindole (DAPI, blue nuclear staining) at 24 h post-I/R (or sham) (original magnification 630x) **(C)** are demonstrated.

Fcgr2b-/- mice spontaneously developed full-blown lupus nephritis (impaired renal function, proteinuria, and increased anti-dsDNA) at 40 weeks old (**Supplementary Figures 1A–C**) without polyarthritis and serositis (data not shown). The renal I/R was performed in 8-week-old Fcgr2b-/- mice, asymptomatic lupus prone condition, and age-matched WT mice demonstrated that there was a similar kidney injury between Fcgr2b-/- and WT mice at 24 h post-renal I/R. Consequently, blood urea nitrogen (BUN), serum creatinine (Scr), proteinuria (UPCI), renal histology (**Figures 1A–E**), and gene expression of cytokines (*TNF-α*, *IL-6* and *IL-10*) in renal tissue (**Supplementary Figures 1D–F**) have no the difference between Fcgr2b-/- and WT mice. Notably, the highest renal injury occurred at 24 h post-renal I/R of both mouse strains supporting a previous publication (28). The remote organ injury at 24 h post-renal I/R has the similarity between both mouse strains, including liver enzyme (serum alanine transaminase), lung injury score, and serum cytokines (**Figures 1F–J**), except for gene expression of cytokines in liver, heart, and lung (**Supplementary Figures 1G–O**). However, prominent NETosis in Fcgr2b-/- mice was demonstrated as follows, i) peripheral blood neutrophils were detected using nuclear morphology (DAPI), which were co-stained with MPO and NE, as well as CitH3 staining, and gene expression of *PAD4* and *IL-1β* (**Figures 2A–H**), ii) serum (serum IL-1β and serum dsDNA) (**Figures 2I, J**), iii) glomeruli were visualized using co-staining of MPO and NE with *PAD4* and *IL-1β* expression (**Figures 3A–D**), CitH3 staining and IL-1β in renal tissue (**Figures 4A–C**), iv) lungs were performed using co-staining of MPO and NE, *PAD4* and *IL-1β* expression (**Supplementary Figures 2A–D**), except liver and heart (**Supplementary Figures 2E–J**).

**Figure 5:**
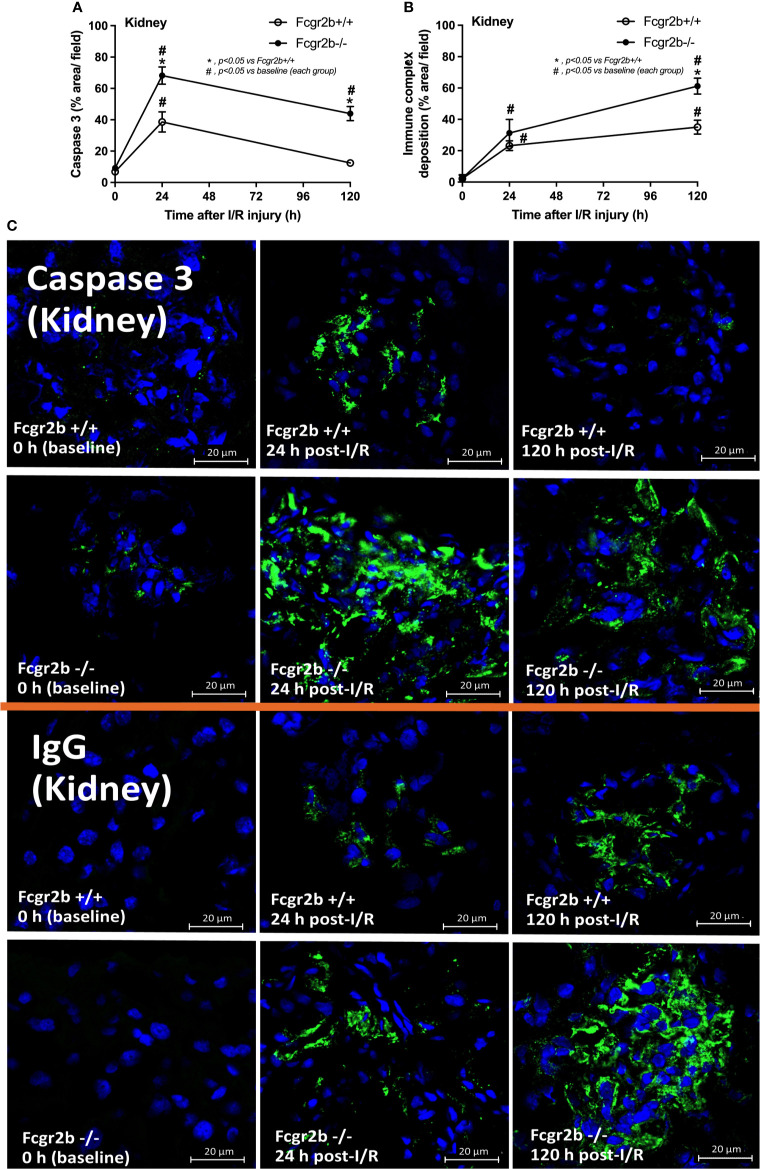
Renal ischemia reperfusion injury (I/R) induced more prominent apoptosis at 24 h post-I/R and immunoglobulin G (IgG) deposition at120 h post-I/R in renal glomeruli of lupus prone mice. Characteristics of renal injury from Fcgr2b-/- or wild-type (Fcgr2b+/+) mice after renal ischemia reperfusion injury (I/R) as evaluated by glomerular apoptosis (activated caspase 3) **(A)**, glomerular IgG deposition **(B)** (n = 6–7/time-point) with the representative immunofluorescent glomerular pictures (activated caspase 3 and IgG deposition) from mice at 0, 24, and 120 h post-I/R (original magnification 630x) **(C)** are demonstrated.

The negative results of NETosis in liver and heart at 24 h post-renal I/R (**Supplementary Figures 1G–L**) suggested that the NETs inflammatory pathways in the remote organ injury post-renal I/R have not occurred. In parallel, apoptosis in glomeruli and lungs, excluding the immunoglobulin (Ig) deposition, was more prominent in Fcgr2b-/- mice compared with WT at 24 h post-renal I/R (**Figures 5A–C** and **Supplementary Figures 3A–C**). These data imply a prominent cell injury (apoptosis and NETosis) in Fcgr2b-/- mice than WT at 24 h post-renal I/R, whereas the functional renal damage was not different.

### Recovery of Neutrophil Extracellular Traps (NETs) and Apoptosis at 120 h Post-Renal I/R With Enhanced Anti-dsDNA and Immunoglobulin Deposition in Fcgr2b-/- Lupus Mice

At 120 h post-renal I/R, renal function (BUN and Scr), non-renal organ damage, and serum cytokines, excepting proteinuria (UPCI), were returned to the baseline level in WT mice (**Figures 1A–I**). Notably, the sustained proteinuria that has a normal renal excretory function (BUN and Scr) supports the renal ischemia-induced podocyte injury (60, 61). In contrast, all the organ injury and serum cytokines in Fcgr2b-/- mice highly increased at 120 h post I/R (**Figures 1A–I** and **Supplementary Figures 1D–P**) with elevated anti-dsDNA (**Figure 1J**), suggesting an exacerbation of lupus disease activity. The rapid elevation of anti-dsDNA in 8-week-old Fcgr2b-/- lupus prone mice after 120 h post-I/R suggesting the non-specific activation of autoreactive B cells in Fcgr2b-/- mice post-renal I/R that was similar to a previous publication (62). Indeed, plasma cells (CD138 and B220 positive cells) and activated B cells (MHC class II and B220 positive cells), except germinal center B cells (CD19 and GL7 positive cells) and follicular B cells (CXCR5 and B220 positive cells), in Fcgr2b-/- mice spleen increased rapidly when compared with WT at 120 h post-renal I/R injury (**Supplementary Figures 4A–E**) that might be responsible for the rapid elevation of anti-dsDNA at 5 days post-renal I/R. Notably, the plasma cells and activated B cells in spleen of Fcgr2b-/- mice and WT were not different from sham control groups (**Supplementary Figures 4A, B**), implied that B cell activation in Fcgr2b-/- mice more rapid than WT, despite a similar B cells baseline state. In addition, NETs of both mouse strains were undetectable at 120 h post-renal I/R (**Figures 2A–J**, **3A–D**, **4A–C** and **Supplementary Figures 2A–J**) with glomerular apoptosis in Fcgr2b-/- mice, in contrast to WT (**Figures 5A–C**). Meanwhile, glomerular IgG deposition was detectable in both mouse strains with more prominent in Fcgr2b-/- mice at 120 h post-renal I/R (**Figures 5B, C**). The IgG deposition at 120 h post I/R, which was a part of the wound healing process (63), was more profound in Fcgr2b-/- mice than WT, partly indicated an increased antibody production from ischemia induced inflammation (64) in lupus mice. These data suggested that prominent NETs and apoptosis (16) at 24 h post-renal I/R enhanced lupus disease exacerbation (12), and increased the auto-antibody production at 120 h post-renal I/R.

### Prominent Apoptosis and Neutrophil Extracellular Traps (NETs) in Fcgr2b-/- Neutrophils, Compared With WT Cells, at 2 h and 4 h of the Stimulation, Respectively, and an Impact of Spleen Tyrosine Kinase (Syk) Signaling

**Figure 6:**
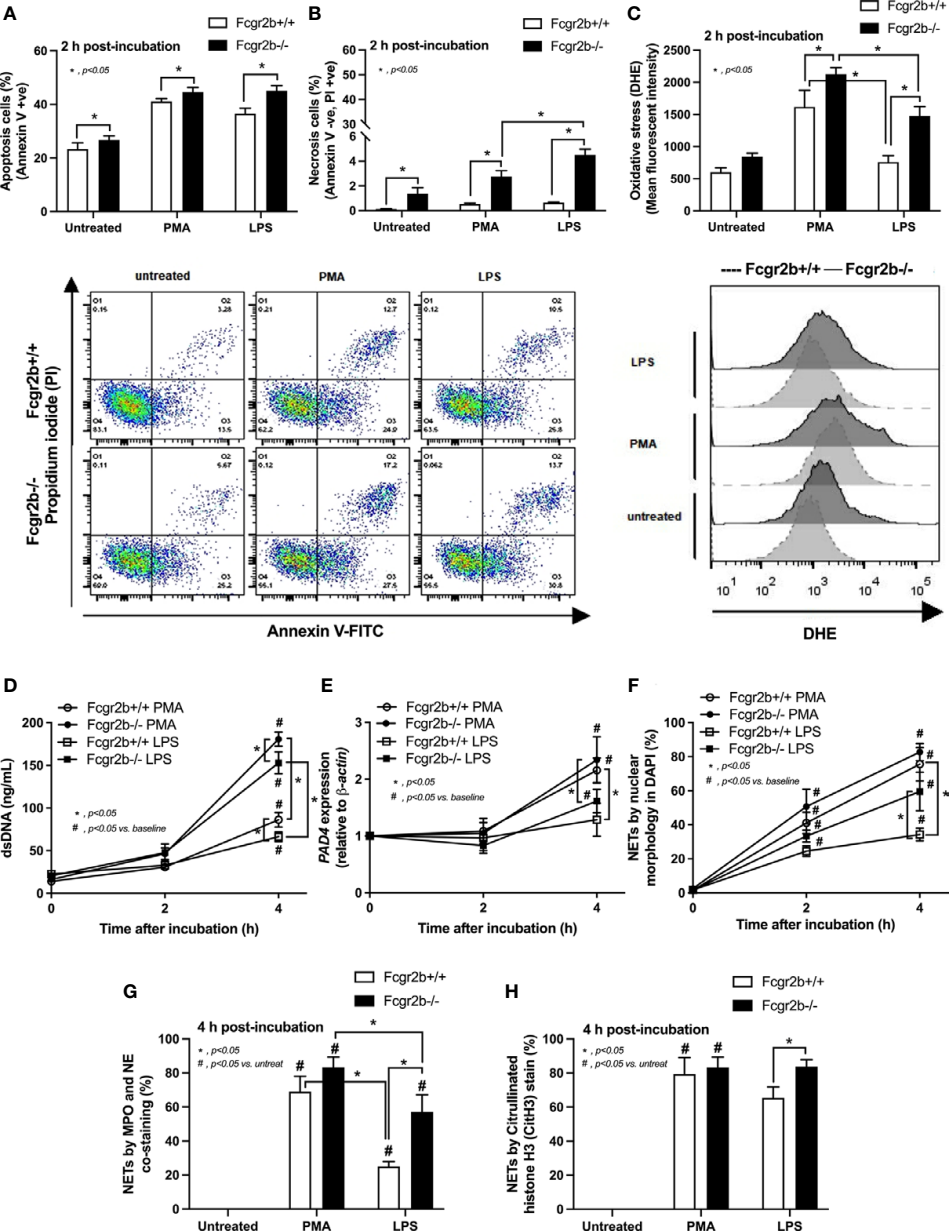
Prominent apoptosis (with oxidative stress) and neutrophil extracellular traps (NETs) at 2 h and 4 h of the stimulation in neutrophils of lupus prone mice. Responses of Fcgr2b-/- and wild-type (Fcgr2b+/+) neutrophils after 2 h activation by phorbol myristate acetate (PMA), which is a NETs activator, or lipopolysaccharide (LPS), which is a TLR-4 stimulator, as evaluated using i) cell apoptosis, positive (+ve) Annexin V with negative (-ve) Propidium iodide (PI) or necrosis, -ve Annexin V negative with +ve PI, with the representative flow cytometry patterns **(A, B)** and ii) reactive oxygen species (ROS) using dihydroethidium (DHE) with the representative flow cytometry patterns **(C)** are demonstrated. Additionally, NETs were determined in time-points by supernatant dsDNA **(D)**, *PAD4* expression **(E)**, nuclear morphology by 4’,6-diamidino-2-phenylindole (DAPI), a blue nuclear staining **(F)** and the co-staining of myeloperoxidases (MPO), and neutrophil elastase (NE) **(G)** or citrullinated histone H3 (CitH3) **(H)** at the 4 h of stimulation using immunofluorescence are demonstrated (independent triplicated experiments were performed).

**Figure 7:**
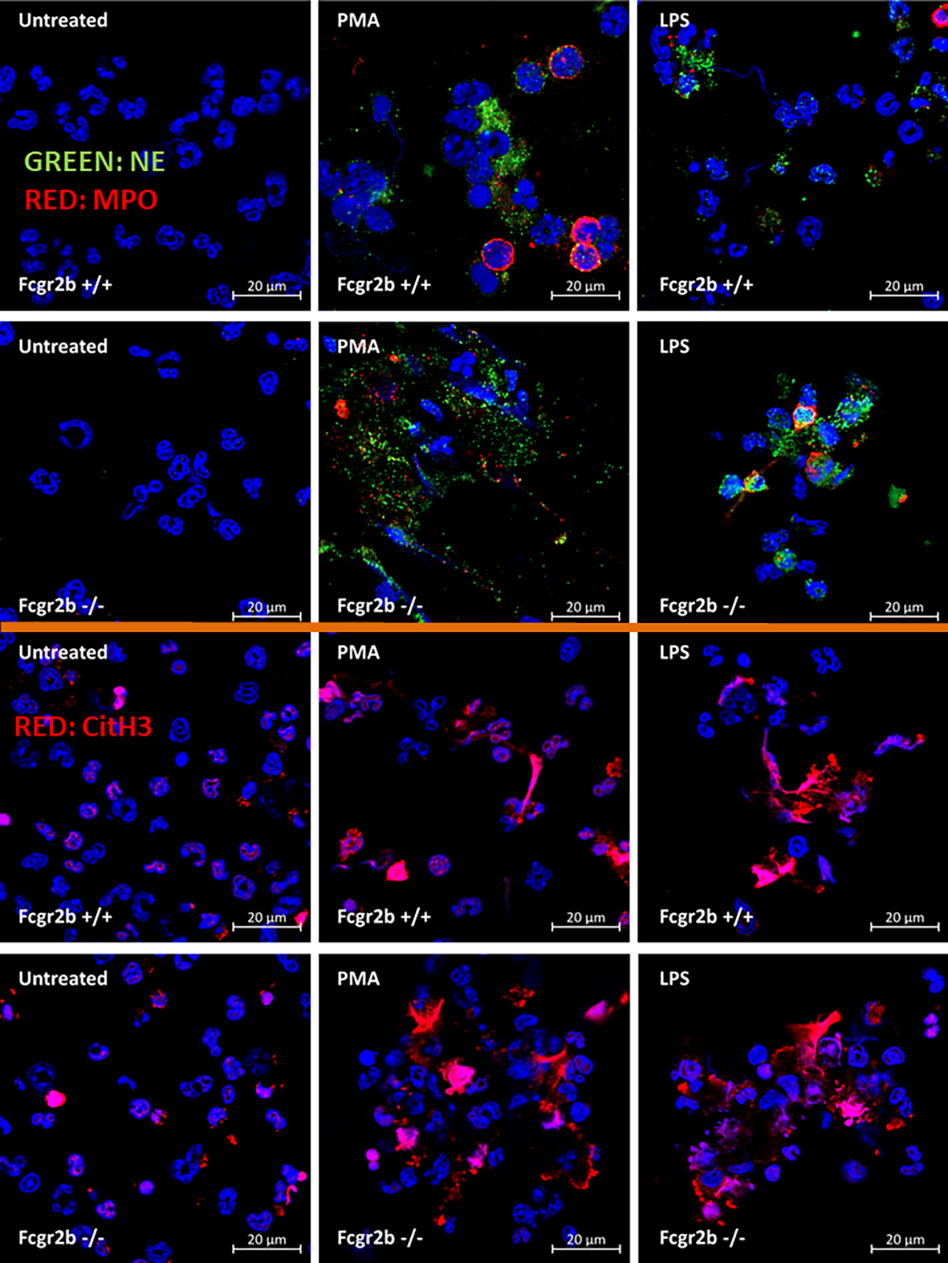
Prominent neutrophil extracellular traps (NETs) at 4 h of the stimulation in neutrophils of lupus prone mice. The representative immunofluorescent pictures of NETs formation in Fcgr2b-/- and wild-type (Fcgr2b+/+) neutrophils after 4 h activation by phorbol myristate acetate (PMA), a NETs activator, or lipopolysaccharide (LPS), a TLR-4 stimulator, as evaluated by the co-staining of myeloperoxidases (MPO) and neutrophil elastase (NE) (upper part) or citrullinated histone H3 (CitH3) (lower part) are demonstrated.

**Figure 8:**
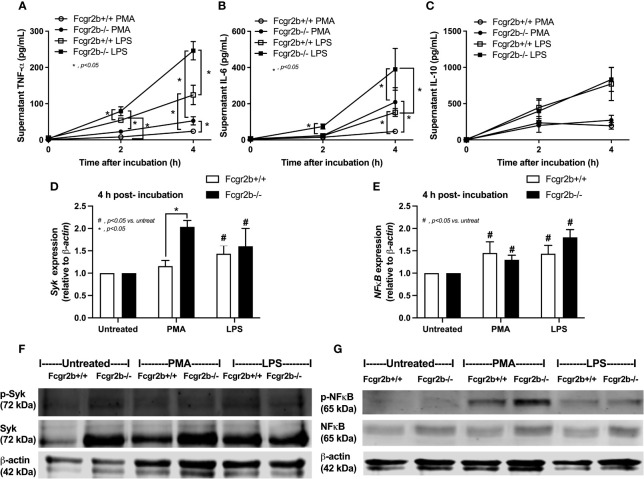
Prominent cytokine production at 4 h through Spleen tyrosine kinase (*Syk*) and nuclear factor kappa B (*NFκB*) in neutrophils of lupus prone mice. Responses of Fcgr2b-/- and wild-type (Fcgr2b+/+) neutrophils after activation by phorbol myristate acetate (PMA), a NETs activator, or lipopolysaccharide (LPS), a TLR-4 stimulator, as evaluated in time-points by supernatant cytokines (TNF-α, IL-6 and IL-10) **(A–C)** and the downstream signals (at 4 h of the stimulation) in gene expression (*Syk* and *NFκB*) **(D, E)** and protein abundances (Syk/phosphorylated-Syk and NFκB/phosphorylated NFκB) with the representative pictures of Western blot analysis **(F, G)** are demonstrated (independent triplicated experiments were performed).

Because i) both apoptosis and NETosis exacerbate lupus disease activity (35), ii) both PMA and LPS could activate apoptosis and NETs (65–67), and iii) Fcgr2b-/- neutrophils might be more susceptible to apoptosis and NETosis than WT cells (the loss of inhibitory signaling) (20, 21), PMA and LPS are used as the representative stimulators. After the 2 h stimulation by PMA or LPS, Fcgr2b-/- neutrophils demonstrated more prominent apoptosis (and necrosis) than WT cells (**Figures 6A, B**), accompanying with the increased production of ROS (Dihydroethidium; DHE) (**Figure 6C**). Furthermore, LPS induced more severe necrosis with lower ROS in Fcgr2b-/- neutrophils when compared with PMA (**Figures 6B, C**). At 4 h of the stimulation, NETosis occurred in neutrophils from both mouse strains as indicated using dsDNA, *PAD4* expression, DAPI nucleus morphology, and co-staining of MPO and NE (**Figures 6D–H**). However, PMA similarly induced NETs in neutrophils of both mouse strains at 4 h post-stimulation, while LPS inducing higher NETs (**Figures 6G, H**, **7**) with the prominent pro-inflammatory cytokine production (TNF-α and IL-6) (**Figures 8A–C**) in Fcgr2b-/- than WT cells. Because spleen tyrosine kinase (Syk) and nuclear factor kappa B (NFκB), a downstream signaling of Syk (68), are the possible shared downstream signaling of PMA and LPS (20, 21, 69, 70), these molecules are explored. At 4 h post-stimulation, PMA upregulated *Syk* only in Fcgr2b-/- neutrophils, while LPS upregulated *Syk* in neutrophils of both mouse strains (**Figures 8D, E**). In parallel, both PMA and LPS similarly upregulated *NFκB* in neutrophils of both mouse strains (**Figures 8D, E**). Meanwhile, both stimulators (PMA and LPS) induced the higher abundance of phosphorylated-Syk and phosphorylated-NFκB in Fcgr2b-/- neutrophils when compared with WT cells (**Figures 8F, G**). As such, detection of NETs (**Figures 6D–H**, **7**) along with Syk and NFκB (gene expression and protein abundance) (**Figures 8A–G**) at 4 h post-stimulation by PMA and LPS implied the association between NETosis and Syk or NFκB, especially in Fcgr2b-/- neutrophils.

### Inhibition of Neutrophil Extracellular Traps (NETs) by Inhibitors Attenuated Renal I/R-Induced Lupus Exacerbation

**Figure 9:**
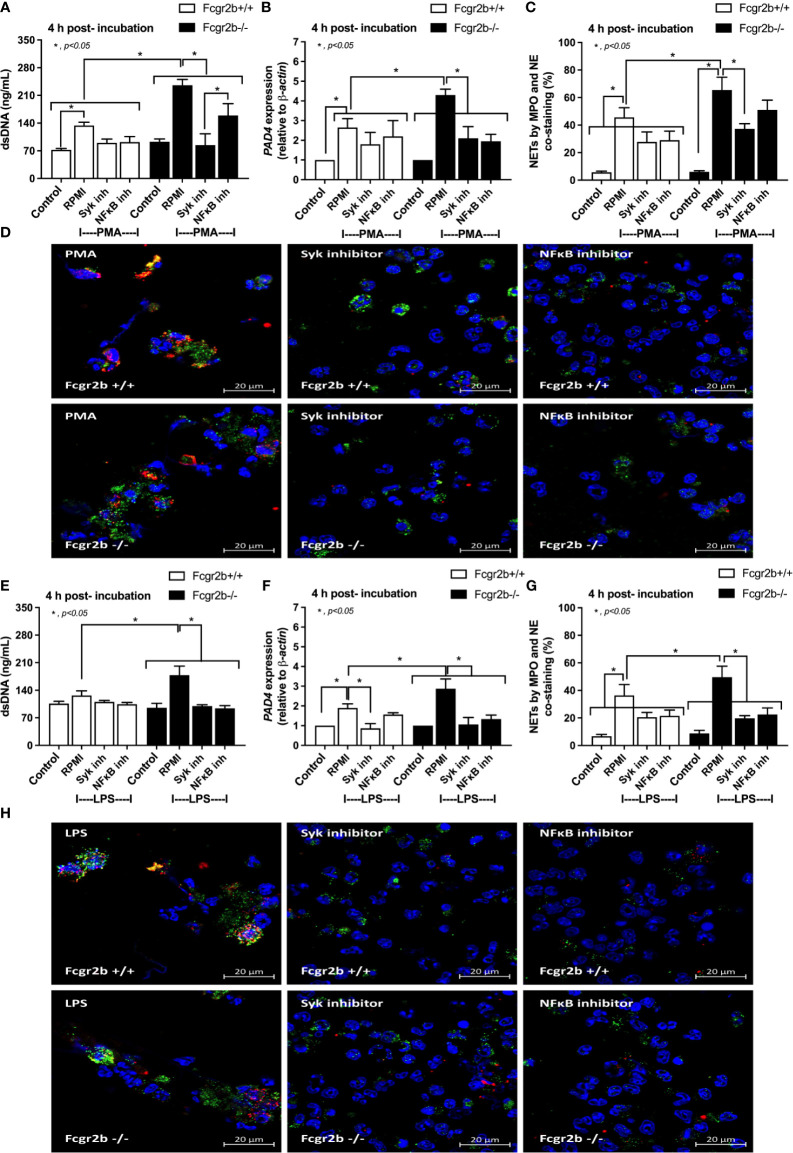
The inhibitors against Spleen tyrosine kinase (Syk) or Nuclear factor kappa B (NFκB) attenuated neutrophil extracellular traps (NETs). NETs formation in Fcgr2b-/- and wild-type (Fcgr2b+/+) neutrophils after 4 h activation by phorbol myristate acetate (PMA), a NETs activator, with or without inhibitors against Syk (Syk inhibitor) or NFκB (NFκB inhibitor) as determined by supernatant dsDNA **(A)**, *PAD4* expression **(B)** and co-staining of myeloperoxidase (MPO) and neutrophil elastase (NE) with the representative immunofluorescent pictures **(C, D)** are demonstrated. In parallel, NETs formation after activation by lipopolysaccharide (LPS), a TLR-4 stimulator, with or without the inhibitors as determined by the same parameters **(E–H)** are also demonstrated (independent triplicated experiments were performed). RPMI, Roswell Park Memorial Institute media (for neutrophils); Control group using only RPMI without the stimulation.

**Figure 10:**
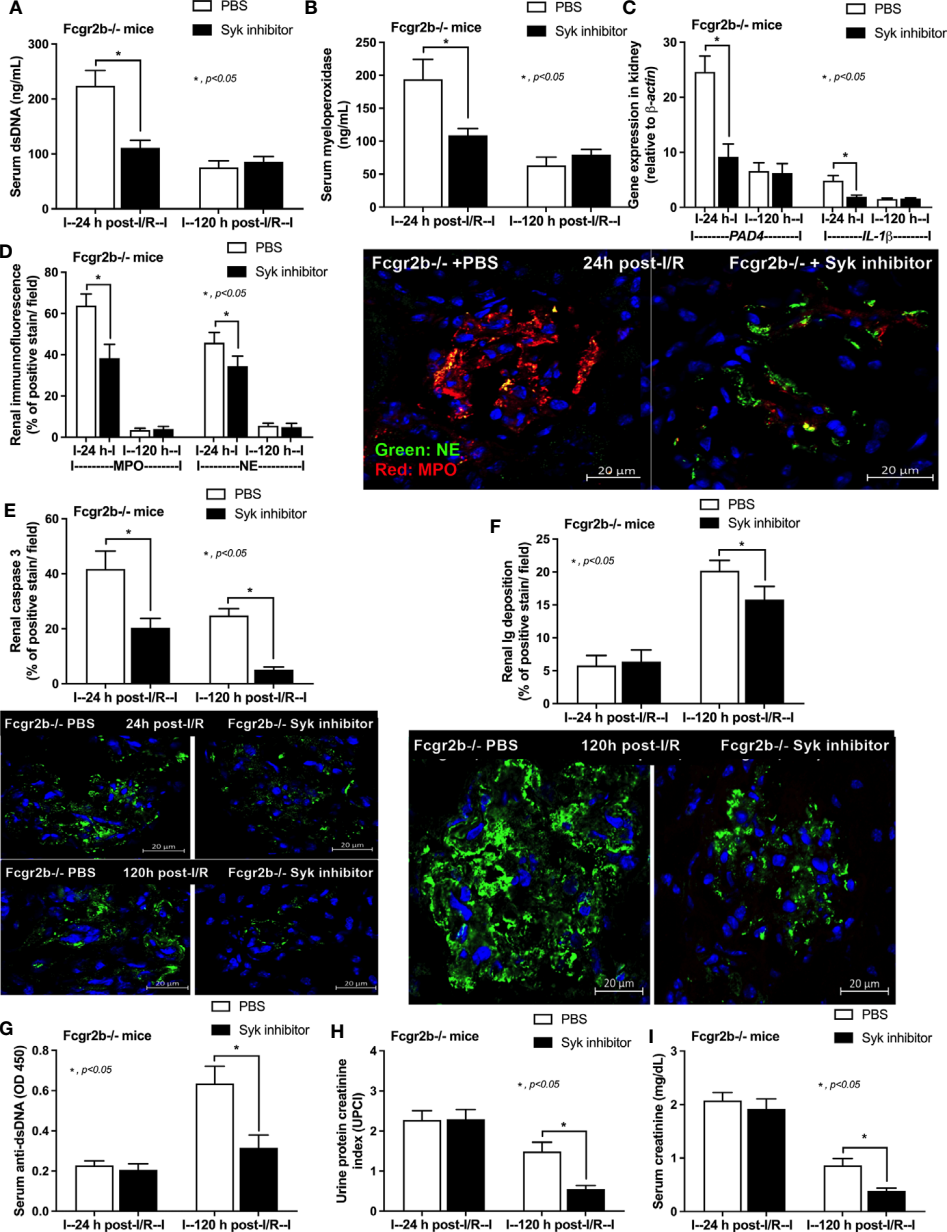
Spleen tyrosine kinase (Syk) inhibitor decreased neutrophil extracellular traps (NETs) at 24 h after renal ischemia reperfusion injury (I/R) and attenuated lupus characteristics (anti-dsDNA, glomerular immunoglobulin deposition and lupus nephritis) at 120 h post-I/R in lupus prone mice. Characteristics of Fcgr2b-/- mice after renal I/R with Syk inhibitor or phosphate buffer solution (PBS) as determined by i) indicators of NETs in serum; serum dsDNA and serum myeloperoxidase (MPO) **(A, B)**, ii) NETs in kidneys; expression of *PAD4* and *IL-1β* **(C)** and co-staining of MPO and neutrophil elastase (NE) with the representative pictures **(D)**, iii) glomerular apoptosis (active caspase 3 staining) and immunoglobulin deposition with the representative pictures **(E, F)**, and iv) lupus nephritis characteristics; serum dsDNA, proteinuria (urine protein creatinine index; UPCI), and serum creatinine **(G–I)** are demonstrated (n = 5–7/group).

**Figure 11:**
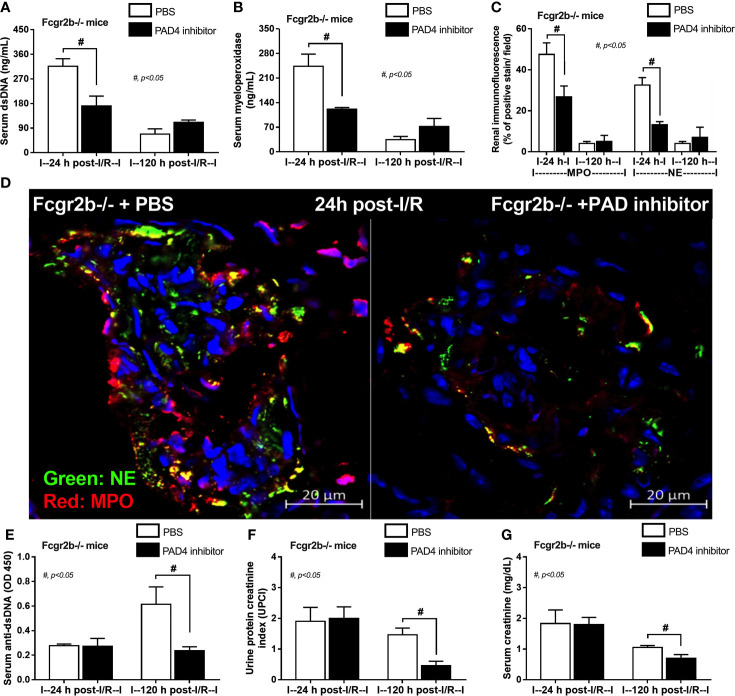
Peptidylarginine deiminase 4 (PAD4) inhibitor decreased neutrophil extracellular traps (NETs) at 24 h after renal ischemia reperfusion injury (I/R) and attenuated lupus characteristics (anti-dsDNA, glomerular immunoglobulin deposition and lupus nephritis) at 120 h post-I/R in lupus prone mice. Characteristics of Fcgr2b-/- mice after renal I/R with PAD4 inhibitor or phosphate buffer solution (PBS) as determined by indicators of NETs in serum; serum dsDNA and serum myeloperoxidase (MPO) **(A, B)**, NETs in kidneys; co-staining of MPO and neutrophil elastase (NE) with the representative pictures **(C, D)** and characteristics of lupus nephritis; serum dsDNA, proteinuria (urine protein creatinine index; UPCI), and serum creatinine **(E–G)** are demonstrated (n = 4–5/group).

Due to the possible Syk and NFκB mediated NETs after PMA and LPS stimulation, inhibitors against Syk and NFκB were tested using the *in vitro* experiments. As such, both inhibitors attenuated i) NETosis by PMA or LPS, which was evaluated by dsDNA, *PAD4* expression and co-detection of MPO with NE (**Figures 9A–H**) and ii) cytokine production (TNF-α, IL-6 and IL-10) (**Supplementary Figures 5A–F**) in neutrophils of both mouse strains (more prominent response to Fcgr2b-/- neutrophils than WT). Because of the NETosis attenuation of Syk inhibitor (39, 71) and the clinically availability of this drug (42) different from other NETs inhibitors, Syk inhibitor was further tested in mice. As such, Syk inhibitor attenuated NETosis (serum dsDNA, MPO, and glomerular NETs) and glomerular apoptosis (**Figures 10A–E**) in Fcgr2b-/- mice at 24 h post renal-I/R affected to decreased glomerular Ig deposition and attenuated lupus activity (anti-dsDNA, proteinuria and Scr) after 120 h post renal-I/R (**Figures 10F–I**). These data indicated that the attenuation of program cell death (apoptosis and NETosis) in renal injury by the Syk inhibitor could prevent lupus disease exacerbation. Although the reduced NETs along with the decreased anti-dsDNA by Syk inhibitor implied an association between NETs and auto-antibody production of post-renal I/R, Syk inhibitor might directly block the antibody production through FcgR signaling (20, 21), except for NETs attenuation. To further support the association between NETs formation and auto-antibody production, a PAD4 inhibitor, a direct NETs inhibitor, was administered in Fcgr2b-/- mice with renal I/R. Indeed, PAD4 inhibitor decreased NETs (serum dsDNA, serum MPO, and glomerular NETs) at 24 h post-renal I/R and attenuated lupus characteristics (anti-dsDNA, proteinuria and Scr) at 120 h post-renal I/R (**Figures 11A–G**) supporting an impact of NETs formation on anti-dsDNA production and lupus exacerbation.

## Discussion

Renal injury at 24 h post renal-I/R prominently induced neutrophil extracellular traps (NETs) and apoptosis in kidneys of Fcgr2b-/- lupus mice that exacerbated the lupus activity at 120 h post renal-I/R. However, the inhibitors against Syk and PAD4 attenuated the NETs at 24 h post-renal I/R and prevented lupus exacerbation at 120 h post-renal I/R.

### Program Cell Death Exacerbates Lupus Disease Activity

To avoid an influence of lupus-induced renal impairment on the renal-I/R model, 8-week-old Fcgr2b-/- mice (asymptomatic lupus prone mice) were used in all experiments and lupus disease activity parameters (anti-dsDNA, proteinuria and Scr) were observed (72). They are well-known for apoptosis (with secondary necrosis), NETosis, and apoNETosis, the pathways of program cell death that cause exposure of auto-antigens from nuclei, which could exacerbate lupus activity (35). Indeed, both of NOX2-dependent NETosis and caspase 3-associated apoptosis after renal-I/R (34, 73) as well as apoNETosis (NOX2-independent NETosis together with caspase 3 activation in the same neutrophils) were activated by high intensity of ultra-violet light (16) might enhance lupus disease activity. Although a simultaneous detection of apoptosis and NETosis at 24 h post renal-I/R was demonstrated, these characteristics was not the apoNETosis (apoptosis and NETosis in the same neutrophils). Perhaps, glomerular apoptosis at 24 h post renal-I/R might be consisted of apoptosis in both renal parenchymal cells and neutrophils. The exploration in this topic is outside the scope of this study. Nevertheless, the enhanced exposure of self-antigens through any cell death pathways possibly exacerbates lupus disease activity (35).

### Prominent NETs and Apoptosis in Fcgr2b-/- Lupus Mice at 24 h Post-Renal I/R

Both NETosis and apoptosis in several cells after renal I/R have been previously mentioned (34, 73, 74), which might be associated with lupus disease exacerbation. Here, there was a similar kidney damage at 24 h post-renal I/R between Fcgr2b-/- and WT mice, but NETs formation (in peripheral blood neutrophils and the internal organs) and apoptosis (in kidneys and lungs) were more prominent in Fcgr2b-/- mice. These data supported the more susceptibility to NETs and apoptosis of Fcgr2b-/- mice than the WT. For the renal I/R-induced NETs, NETs in neutrophils locally accumulated in the injured kidneys and remotely deposited in the lungs through the induction of damage, which was associated with the Damage Associated Molecular Patterns (DAMPs) from renal tubular necrosis as previously published (75). However, the effect of renal I/R on Fcgr2b-/- mice was more prominent than WT mice perhaps through the hyper-responsiveness from inhibitory Fc gamma receptor (FcgR) deficiency (76). The upregulation of activating-FcgRs without Fcgr2b inhibitory signaling partly generated the increased responses of Fcgr2b-/- macrophages (8, 77). Because i) NETosis from activation on TLR-4, one of the receptors for DAMPs (78, 79), is well-known (80), ii) the crosstalk between TLR-4 and activating-FcgRs (20, 21, 59), which is the profound reaction against DAMPs of Fcgr2b-/- mice, is possible, and iii) both TLR-4 and FcgRs are presented on neutrophils (78, 81). Therefore, the more severe NETosis at 24 h post-renal I/R in Fcgr2b-/- mice is probably resulted from the TLR-4/activating-FcgRs cross-talk on neutrophils, which is similar to the Fcgr2b-/- macrophages (20, 21). However, renal ischemia induced liver and cardiac injury (27, 28) was not associated with NETs that would be consistent with the non-detectable NETs in these organs. Notably, the upregulated *PAD4* and *IL-1β* without the positive detection of MPO and NE in livers and hearts supported pro-inflammatory activation (27, 28) in these organs without NETosis.

On the other hand, the renal I/R induces apoptosis of the renal parenchymal cells and the accumulated immune cells (32, 33), especially neutrophils (29–31). At 24 h post-renal I/R, apoptosis in kidneys and lungs of Fcgr2b-/- mice was more prominent than WT mice, possibly due to the hyper-inflammation induced apoptosis. Accordingly, DAMPs-activated TLR-4 induces massive apoptosis in parenchymal cells (kidneys and lungs) and immune cells (macrophages and neutrophils) after renal-I/R is well-known (82–85). Furthermore, the enhanced TLR-4 activation in Fcgr2b-/- mice through TLR-4/activating-FcgRs cross-talk (20, 21), might also be responsible to immune hyper-responsiveness and profound apoptosis. Nevertheless, both NETs and apoptosis were more prominent in Fcgr2b-/- mice than WT at 24 h post-I/R, which possibly enhanced self-antigens presentation and increased auto-antibody production (12, 16).

### Prominent Anti-dsDNA and Immune Complex Deposition, but Not NETs, in Fcgr2b-/- Lupus Mice at 120 h Post-Renal I/R Exacerbates the Lupus Activity

The association between the program cell death pathways and the lupus exacerbation through the enhanced auto-antibody production is well-established (35). Here, the program cell deaths (NETosis and apoptosis) at 24 h post-renal I/R enhanced the production of anti-dsDNA in Fcgr2b-/- mice at 120 h post-renal I/R were demonstrated. The prominent anti-dsDNA, which is a major lupus auto-antibody, in Fcgr2b-/- mice at 120 h post-renal I/R suggests an impact of the loss of immune tolerance in these lupus mice (25). Interestingly, anti-dsDNA is inducible very shortly after I/R (5 days) in 8-week-old Fcgr2b-/- mice (asymptomatic lupus prone mice), despite the low-level of anti-dsDNA at baseline which is similar to the level in WT mice, indicating the existence of auto-reactive B cells in Fcgr2b-/- mice. Indeed, the clonal expansion of self-reactive B cells in germinal center is one of the main pathogenesis of autoimmune diseases (86). In this study, plasma cells and activated B cells in spleen of Fcgr2b-/- mice were higher than WT at 120 h post-renal I/R, supporting that a prominent activity of these immune cells in lupus that might be responsible for the rapid induction of anti-dsDNA. Therefore, the loss of inhibitory FcgRs in autoreactive B cells of Fcgr2b-/- mice might be more susceptible to the non-specific inflammatory activation from renal-I/R, which led to a rapid clonal expansion and an acceleration of the auto-antibody production (62).

Additionally, the prominent immune complex (IC) deposition at 120 h post-renal I/R in Fcgr2b-/- mice possibly resulted in mononuclear cell infiltrations and inflammatory responses in several organs. In contrast, the only abnormality in WT mice at 120 h post-renal I/R was proteinuria (with normal Scr) due to the incomplete recovery of proximal renal tubules (87). At 120 h post-renal I/R, Fcgr2b-/- mice demonstrated the lupus characteristics as indicated by Scr, anti dsDNA, proteinuria, and glomerular IC deposition. Due to the non-detectable renal MPO and NE in lupus mice at 120 h post-I/R, renal injury was not directly caused by NETs but perhaps the enhanced glomerular IC deposition. Hence, profound NETs and apoptosis at 24 h post renal-I/R in Fcgr2b-/- mice further caused the lupus exacerbation at 120 h post-I/R which might be due to i) accelerated auto-antibody production from the enhanced self-antigen presentation by NETosis and apoptosis (88–90) and ii) IC deposition induced inflammation (91). These results indicated that acute kidney injury in lupus acted as an exacerbating factor through the program cell death pathways.

### The Increased Susceptibility to Apoptosis and NETosis of Fcgr2b-/- Neutrophils and the Syk Inhibition: A Potential Clinical Translation

Although the activation of inhibitory-Fcgr2b in T cells could induce the T cell apoptosis (92), the hyper-inflammatory responses of Fcgr2b-/- macrophages (due to the inhibitory loss) cause the profound LPS-induced apoptosis (13). Because the inhibitory Fcgr2b presents in all myeloid cells (93) (not only macrophages but also neutrophils), Fcgr2b-/- neutrophils might be more susceptible to the cell death pathways. Although, PMA (94) and LPS (95) are a well-known NETs stimulator and a potent apoptosis inducer, respectively, both agents could induce both apoptosis and NETosis (65–67, 96, 97). Indeed, PMA and LPS induced the more prominent apoptosis and NETosis through Syk and NFκB in Fcgr2b-/- neutrophils compared with WT at 2 h and 4 h, respectively, supported the previous publications (69, 98). Because the data of NETosis were less compared to apoptosis in Fcgr2b-/- immune cells, NETosis was further explored. As such, both gene expression and protein quantification of Syk and NFκB in Fcgr2b-/- neutrophils were higher than WT cells and the inhibition of both Syk and NFκB attenuated the NETosis, indicating a possible association between Syk and Fc gamma receptors (46, 99). In addition, the association between NETs and lupus exacerbation was demonstrated in renal I/R Fcgr2b-/- mice along with the inhibitors against NETs (Syk and PAD4 inhibitors). Both inhibitors effectively attenuated NETs at 24 h post-renal I/R and decreased anti-dsDNA at 120 h of the model, supporting that NETs blockade could prevent the renal I/R-induced lupus exacerbation.

**Figure 12:**
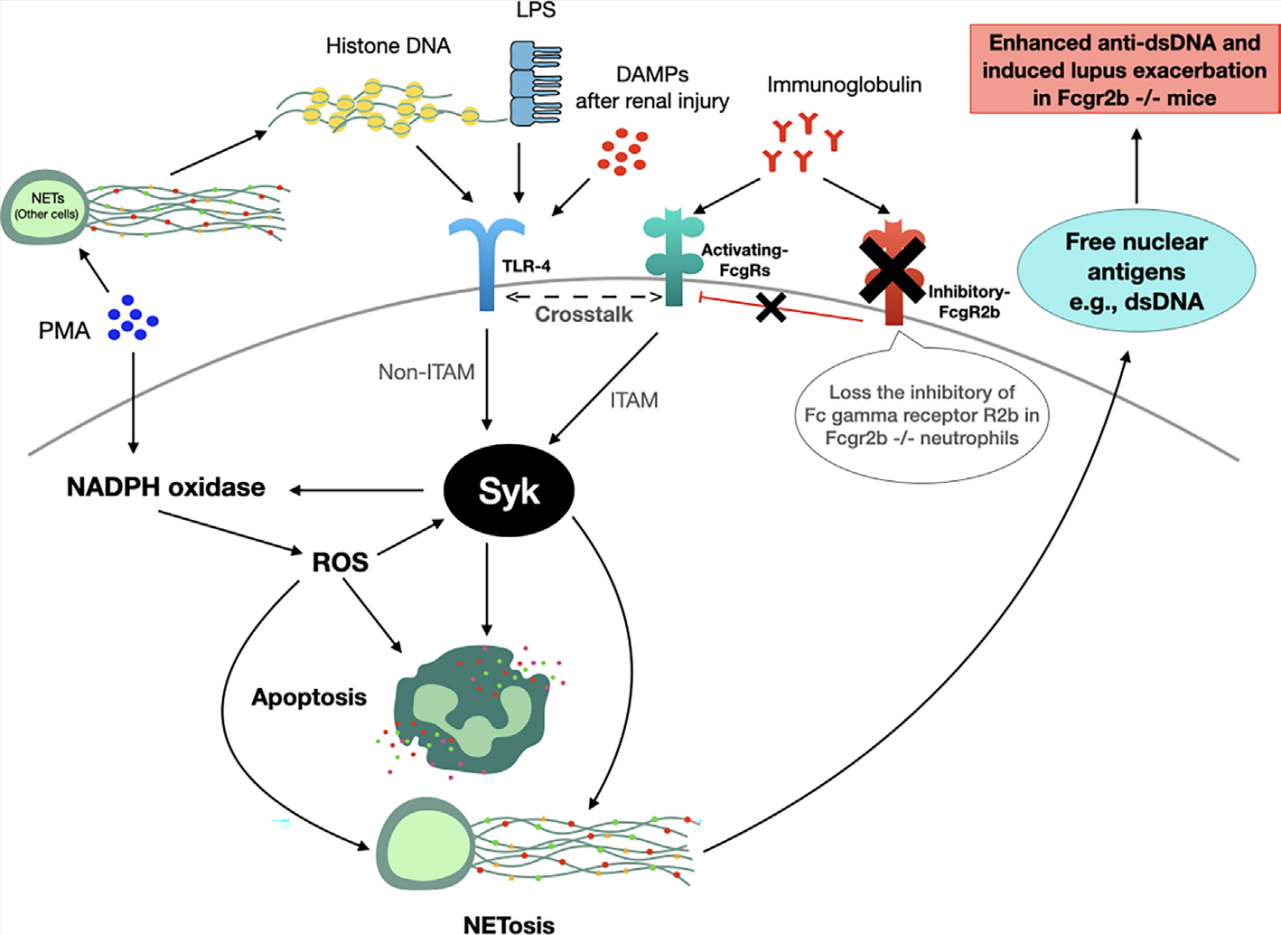
The working hypothesis. Spleen tyrosine kinase (Syk) is a possible shared-downstream signaling of i) reactive oxygen species (ROS) derived from NADPH oxidase 2 (NOX2)-dependent PMA stimulation (100), ii) direct TLR-4 activation through DAMPs (damage associated molecular patterns) from renal ischemia (in mice) or lipopolysaccharide (LPS) (*in vitro*), iii) indirect TLR-4 activation from DNA histone released from PMA-induced NETosis (99) and iv) Fc gamma receptors (FcgRs) (98). Indeed, Syk, activated through immunoreceptor tyrosine-based activation motif (ITAM) and non-ITAM dependent signals from FcgRs and TLR-4, respectively (37), could enhance both apoptosis (2 h post-stimulation) and NETosis (4 h post-stimulation) (100, 101). Hence, prominent Syk activation in Fcgr2b-/- neutrophils compared with wild-type (WT) might be due to the crosslink between TLR-4 and activating-FcgRs (20, 21, 58) without the inhibitory receptor (blue cross line). After that, the profound free nuclear antigens, including dsDNA, from apoptosis and NETosis exacerbates lupus activity from the prominent anti-dsDNA production in Fcgr2b-/- mice but not in WT (+ve, positive signal; -ve, negative signal).

The working hypothesis of the *in vivo* and *in vitro* experiments is presented in **Figure 12**. Accordingly, TLR-4 is an important pathway for apoptosis and NETosis (36), which could be induced by LPS, DAMPs (from renal-I/R), and DNA histones (from NETosis of PMA and renal-I/R) (100) possibly through the NADPH oxidase 2 (NOX2) dependent pathway (66, 102, 103). Likewise, Fc gamma receptors are the initiation of NETosis through Syk activation (37). Because i) Syk is a downstream of Fc gamma receptors and TLR-4 through immunoreceptor tyrosine-based activation motif (ITAM) and non-ITAM dependent pathways, respectively (37), ii) Syk is a possible shared downstream signaling from TLR-4, Fc gamma receptors and PMA (**Figure 10**), which activated the Syk through ROS from NOX2-dependent PMA stimulation (102), and iii) Syk also enhances ROS production (104, 105) that could accelerate both apoptosis and NETosis (102), and this inhibitor is an interesting drug for use in lupus. Thus, a possible mechanism that is responsible for prominent apoptosis and NETosis in Fcgr2b-/- neutrophils over WT might be an enhanced TLR-4 activity due to the crosstalk between TLR-4 and activating Fc gamma receptors (106) through Syk (and NFκB) without the inhibitory Fcgr2b (**Figure 12**). Despite an incomplete data on the association between Syk and Fc gamma receptors, the attenuation of NETosis and apoptosis with the prevention on lupus exacerbation by Syk inhibitor in Fcgr2b-/- mice with renal-I/R is interesting. Since Syk inhibitors are clinically available (107–110) for either lupus (42) or non-lupus conditions (20, 21, 101, 111), Syk inhibitor might be useful for the prevention of renal injury-induced lupus exacerbation. Future studies are of interest.

In conclusion, the prominent NETs and the Syk activation were observed in Fcgr2b-/- mice after renal I/R injury that induced lupus exacerbation. The attenuation of NETs using Syk inhibitor, possibly through reduction of the downstream signaling of TLR-4 and Fc gamma receptors, was proposed as an interesting strategy for the treatment in lupus. Further studies are warranted.

## Data Availability Statement

The datasets presented in this study can be found in online repositories. The names of the repository/repositories and accession number(s) can be found in the article/**Supplementary Material**.

## Ethics Statement

The animal study was reviewed and approved by Institutional Animal Care and Use Committee of the Faculty of Medicine, Chulalongkorn University, Bangkok, Thailand. Written informed consent was obtained from the owners for the participation of their animals in this study.

## Author Contributions

WS designed and coordinated all the experiments, performed *in vitro* and *in vivo* experiments, and wrote and approved the manuscript. SS performed *in vitro* experiments, and approved the manuscript. PPh performed *in vitro* and *in vivo* experiments, and approved the manuscript. KU performed *in vitro* experiments, and approved the manuscript. TB performed *in vitro* experiments, and approved the manuscript. PV performed *in vivo* experiments, and approved the manuscript. AC performed *in vitro* experiments, and approved the manuscript. PPi supervised *in vitro* experiments, and approved the manuscript. DC supervised *in vitro* experiments, and approved the manuscript. AL designed and coordinated all the experiments, analyzed all of these experiment, and wrote and approved the manuscript. All authors contributed to the article and approved the submitted version.

## Funding

This research is supported by the Program Management Unit for Human Resources & Institutional Development Research and Innovation-CU [Global Partnership B16F630071 and Flagship B05F630073], and the TSRI Fund (CU_FRB640001_01_23_1) and National Research Council of Thailand. WS is funded by the 90th Anniversary of Chulalongkorn University Fund (Ratchadapisek Sompote Endowment Fund).

## Conflict of Interest

The authors declare that the research was conducted in the absence of any commercial or financial relationships that could be construed as a potential conflict of interest.

## Supplementary Material

Supplementary Figure 1 The age-dependent lupus characteristics of Fcgr2b-/- mice and the injury from renal ischemia reperfusion injury (I/R). Age-dependent lupus nephritis of Fcgr2b-/- mice compared with wild-type (Fcgr2b+/+) mice; serum creatinine, urine protein creatinine index (UPCI) and serum anti-dsDNA, **(A–C)** and the characteristics in time-point of Fcgr2b-/- or Fcgr2b+/+ mice after renal ischemia reperfusion injury (I/R) as determined by gene expression of cytokines (*TNF-α*, *IL-6* and *IL-10*) in kidneys **(D–F)**, liver **(G–I)**, heart **(J–L)**, lung **(M–O)** and the representative Hematoxylin and Eosin (H&E) staining (original magnification 200x) of liver and lung at 120 h post renal I/R **(P)** are demonstrated (n = 6–7/time-point). Notably, the renal histological pictures at other time-points of both mouse strains are not demonstrated due to the similarity to Fcgr2b-/- mice at 120 h post I/R (normal histology).

Supplementary Figure 2 Renal ischemia reperfusion injury (I/R) induced more prominent neutrophil extracellular traps (NETs) in lungs of lupus prone mice. Characteristics of NETs in lungs from Fcgr2b-/- or wild-type (Fcgr2b+/+) mice after renal ischemia reperfusion injury (I/R) as determined by co-staining of myeloperoxidases (MPO) and neutrophil elastase (NE) at 24 h post-I/R **(A)** (n = 5–8/group) and gene expression in the time-point of *IL-1β* and *PAD4* **(B, C)** (n = 6–7/time-point) with the representative immunofluorescent pictures of NE (green), MPO (red) and 4’,6-diamidino-2-phenylindole (DAPI, blue nuclear staining) at 24 h post-I/R (or sham) (original magnification 630x) **(D)** are demonstrated. Additionally, the score of MPO and NE co-staining at 24 h post-I/R **(E)** with *IL-1β* and *PAD4* in liver **(F, G)** and in hearts **(H–J)** (n = 6–7/group or time-point) are also demonstrated.

Supplementary Figure 3 Renal ischemia reperfusion injury (I/R) induced more prominent apoptosis and immunoglobulin G (IgG) deposition in lungs of lupus prone mice. Characteristics of lung injury from Fcgr2b-/- or wild-type (Fcgr2b+/+) mice after renal ischemia reperfusion injury (I/R) as evaluated by apoptosis (activated caspase 3) **(A)**, IgG deposition **(B)** (n = 6–7/time-point for **A–D)** with the representative immunofluorescent pictures (activated caspase 3 and IgG deposition) from mice at 0, 24 and 120 h post-I/R (original magnification 630x) **(E)** are demonstrated.

Supplementary Figure 4 Prominent activation of plasma cells and activated B cells in spleen of lupus mice at 120 h post-renal I/R. Characteristics of immune cells in spleen at 120 h after sham or renal ischemia reperfusion injury (I/R) from Fcgr2b-/- and wild-type (Fcgr2b+/+) mice as indicated by the abundance in spleen of plasma cell (CD138 and B220 positive cells) **(A)**, activated B cells (MHC II and B220 positive cells) **(B)**, germinal center B cells (CD19 and GL7 positive cells) **(C)**, follicular B cells (CXCR5 and B220 positive cells) **(D)** and the representative flow cytometry analysis of plasma cells and activated B cells **(E)** are demonstrated (n = 4-5/group).

Supplementary Figure 5 Inhibitors against Spleen tyrosine kinase (Syk) or Nuclear factor kappa B (NFκB) attenuated neutrophil cytokine production. Supernatant cytokines (TNF-α, IL-6 and IL-10) in Fcgr2b-/- and wild-type (Fcgr2b+/+) neutrophils after 4 h activation by phorbol myristate acetate (PMA), a NETs activator, with or without inhibitors against Syk (Syk inh) or NFκB (NFκB inh) **(A–C)** or activation by lipopolysaccharide (LPS), a TLR-4 stimulator, **(D–F)** are demonstrated (independent triplicated experiments were performed). RPMI, Roswell Park Memorial Institute media (for neutrophils); Control group using only RPMI without the stimulation.
