# Supplementary figures and images for: Acute Kidney Injury Induced Lupus Exacerbation Through the Enhanced Neutrophil Extracellular Traps (and Apoptosis) in Fcgr2b Deficient Lupus Mice With Renal Ischemia Reperfusion Injury
Source: Front Immunol. 2021 Jun 24;12:669162. doi: 10.3389/fimmu.2021.669162 (PMC8269073; doi:10.3389/fimmu.2021.669162)

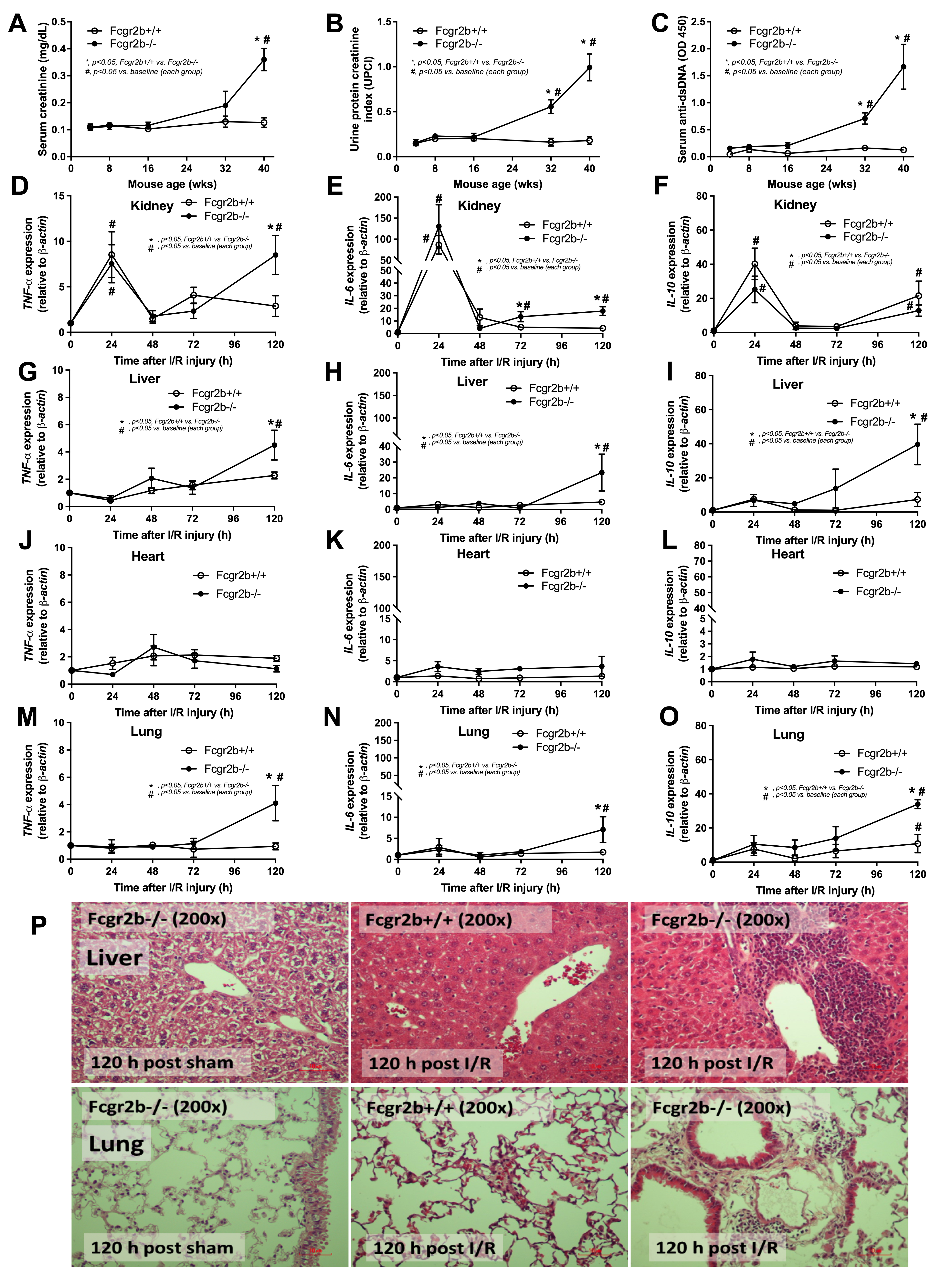

Supplement: Supplementary Figure 1 — The age-dependent lupus characteristics of Fcgr2b-/- mice and the injury from renal ischemia reperfusion injury (I/R). Age-dependent lupus nephritis of Fcgr2b-/- mice compared with wild-type (Fcgr2b+/+) mice; serum creatinine, urine protein creatinine index (UPCI) and serum anti-dsDNA, (A–C) and the characteristics in time-point of Fcgr2b-/- or Fcgr2b+/+ mice after renal ischemia reperfusion injury (I/R) as determined by gene expression of cytokines (TNF-α, IL-6 and IL-10) in kidneys (D–F), liver (G–I), heart (J–L), lung (M–O) and the representative Hematoxylin and Eosin (H&E) staining (original magnification 200x) of liver and lung at 120 h post renal I/R (P) are demonstrated (n = 6–7/time-point). Notably, the renal histological pictures at other time-points of both mouse strains are not demonstrated due to the similarity to Fcgr2b-/- mice at 120 h post I/R (normal histology). [file Image_1.tiff]

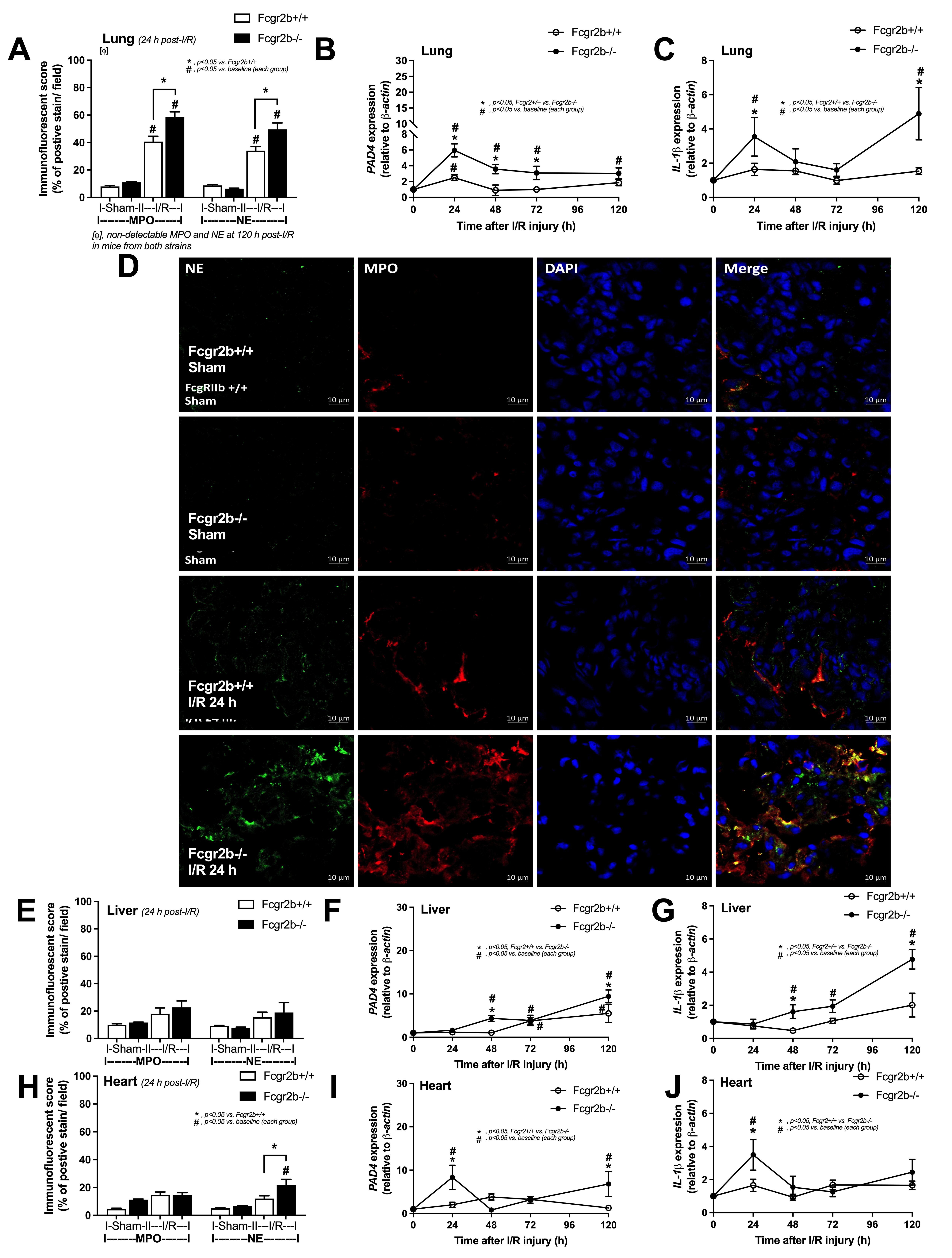

Supplement: Supplementary Figure 2 — Renal ischemia reperfusion injury (I/R) induced more prominent neutrophil extracellular traps (NETs) in lungs of lupus prone mice. Characteristics of NETs in lungs from Fcgr2b-/- or wild-type (Fcgr2b+/+) mice after renal ischemia reperfusion injury (I/R) as determined by co-staining of myeloperoxidases (MPO) and neutrophil elastase (NE) at 24 h post-I/R (A) (n = 5–8/group) and gene expression in the time-point of IL-1β and PAD4 (B, C) (n = 6–7/time-point) with the representative immunofluorescent pictures of NE (green), MPO (red) and 4’,6-diamidino-2-phenylindole (DAPI, blue nuclear staining) at 24 h post-I/R (or sham) (original magnification 630x) (D) are demonstrated. Additionally, the score of MPO and NE co-staining at 24 h post-I/R (E) with IL-1β and PAD4 in liver (F, G) and in hearts (H–J) (n = 6–7/group or time-point) are also demonstrated. [file Image_2.tiff]

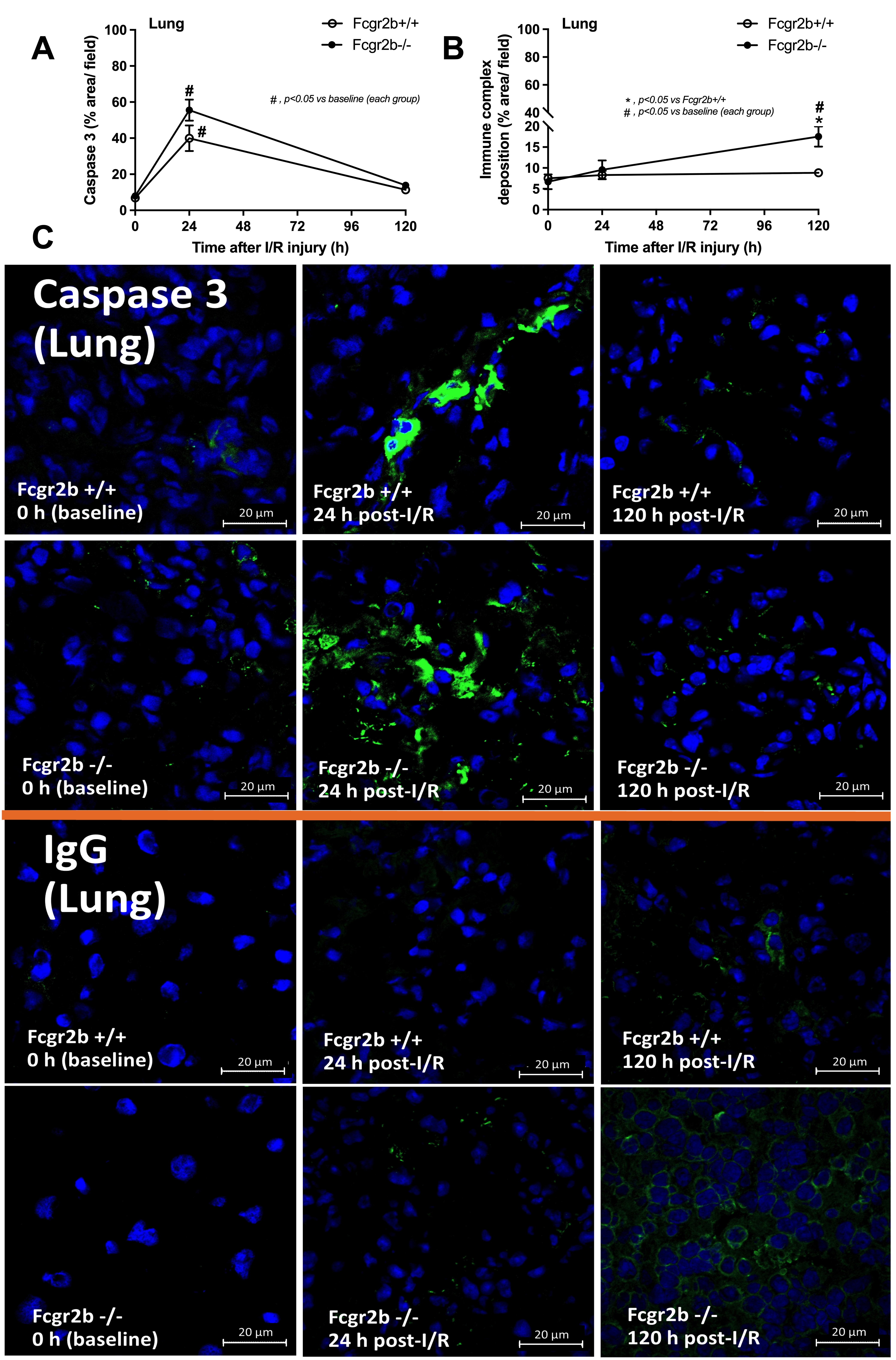

Supplement: Supplementary Figure 3 — Renal ischemia reperfusion injury (I/R) induced more prominent apoptosis and immunoglobulin G (IgG) deposition in lungs of lupus prone mice. Characteristics of lung injury from Fcgr2b-/- or wild-type (Fcgr2b+/+) mice after renal ischemia reperfusion injury (I/R) as evaluated by apoptosis (activated caspase 3) (A), IgG deposition (B) (n = 6–7/time-point for A–D) with the representative immunofluorescent pictures (activated caspase 3 and IgG deposition) from mice at 0, 24 and 120 h post-I/R (original magnification 630x) (E) are demonstrated. [file Image_3.tiff]

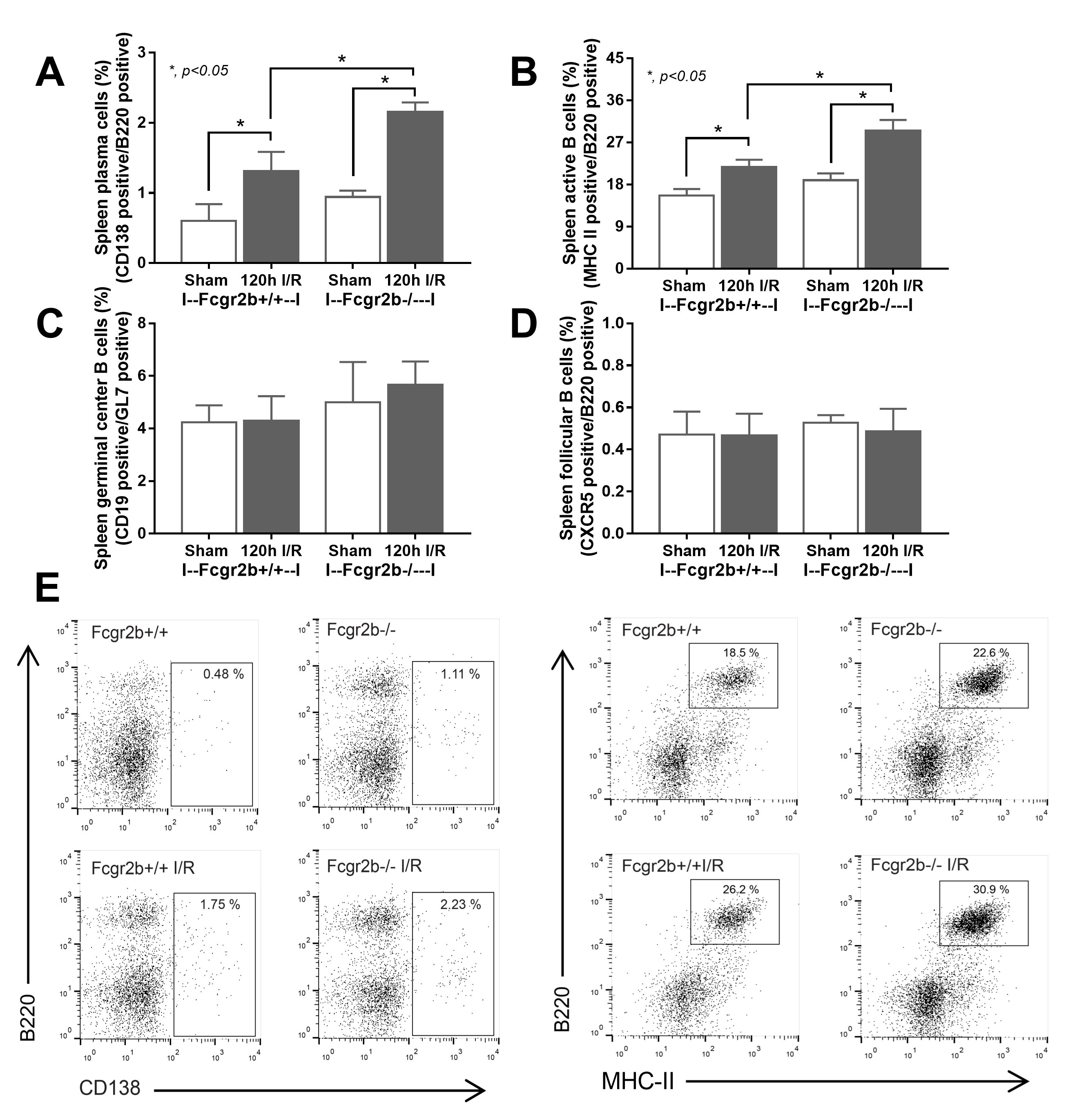

Supplement: Supplementary Figure 4 — Prominent activation of plasma cells and activated B cells in spleen of lupus mice at 120 h post-renal I/R. Characteristics of immune cells in spleen at 120 h after sham or renal ischemia reperfusion injury (I/R) from Fcgr2b-/- and wild-type (Fcgr2b+/+) mice as indicated by the abundance in spleen of plasma cell (CD138 and B220 positive cells) (A), activated B cells (MHC II and B220 positive cells) (B), germinal center B cells (CD19 and GL7 positive cells) (C), follicular B cells (CXCR5 and B220 positive cells) (D) and the representative flow cytometry analysis of plasma cells and activated B cells (E) are demonstrated (n = 4-5/group). [file Image_4.tif]

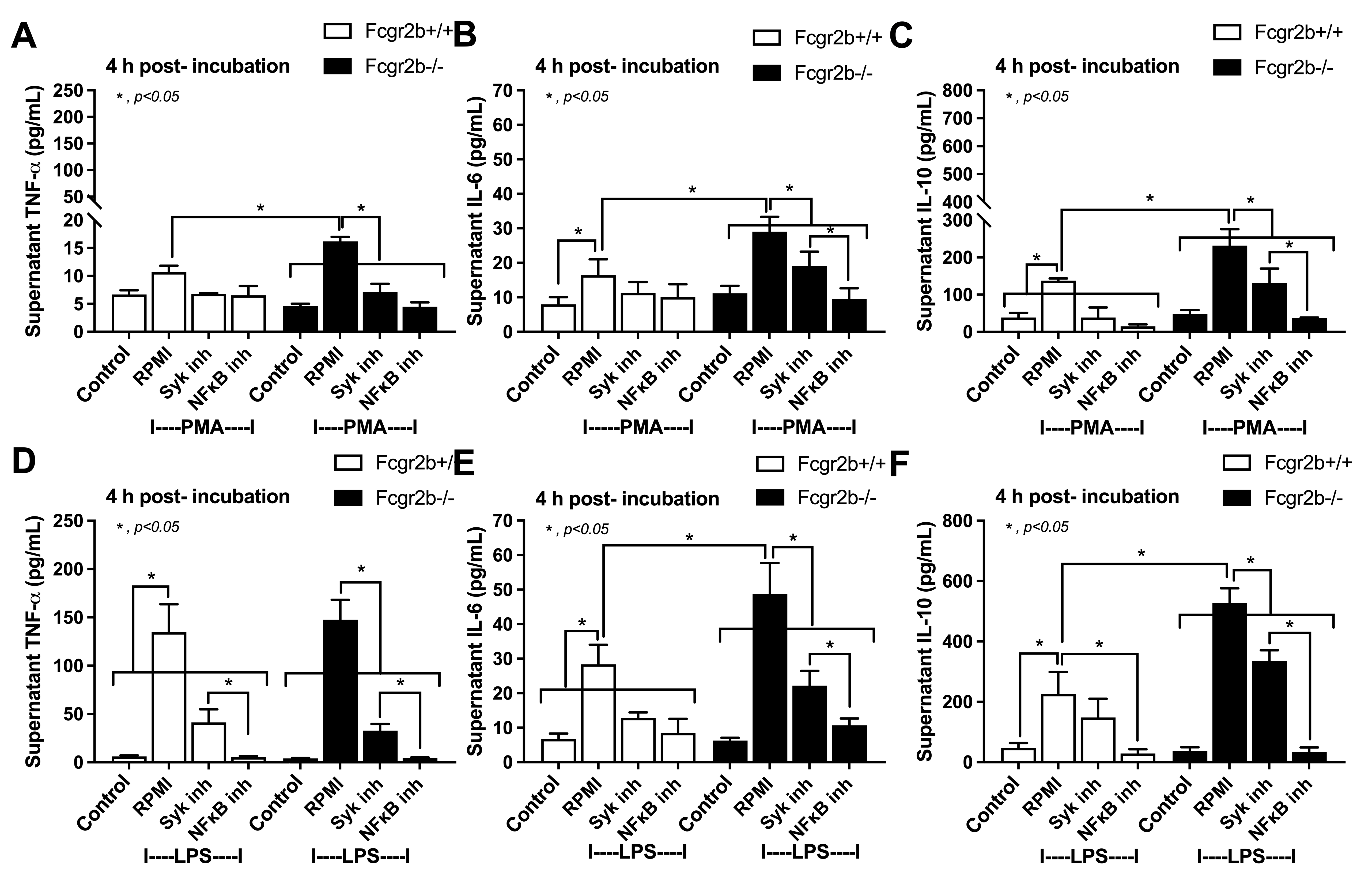

Supplement: Supplementary Figure 5 — Inhibitors against Spleen tyrosine kinase (Syk) or Nuclear factor kappa B (NFκB) attenuated neutrophil cytokine production. Supernatant cytokines (TNF-α, IL-6 and IL-10) in Fcgr2b-/- and wild-type (Fcgr2b+/+) neutrophils after 4 h activation by phorbol myristate acetate (PMA), a NETs activator, with or without inhibitors against Syk (Syk inh) or NFκB (NFκB inh) (A–C) or activation by lipopolysaccharide (LPS), a TLR-4 stimulator, (D–F) are demonstrated (independent triplicated experiments were performed). RPMI, Roswell Park Memorial Institute media (for neutrophils); Control group using only RPMI without the stimulation. [file Image_5.tiff]
