# Supplementary material for: Acute Kidney Injury Induced Lupus Exacerbation Through the Enhanced Neutrophil Extracellular Traps (and Apoptosis) in Fcgr2b Deficient Lupus Mice With Renal Ischemia Reperfusion Injury
Source: Front Immunol. 2021 Jun 24;12:669162. doi: 10.3389/fimmu.2021.669162 (PMC8269073; doi:10.3389/fimmu.2021.669162)
Supplement: Supplementary file 6 [file Table_1.pdf]

Supplementary Table 1. List of Primers in the study are demonstrated.

**Primers**	**Sequence**	
Tumor necrosis factor-α (*TNF-a*)	**Forward**	5’-CCTCACACTCAGATCATCTTCTC-3’
	**Reverse**	5’-AGATCCATGCCGTTGGCCAG-3’
Interleukin-6 (*IL-6*)	**Forward**	5’-TACCACTTCACAAGTCGGAGGC-3’
	**Reverse**	5’-CTGCAAGTGCATCATCGTTGTTC-3’
Interleukin-10 (*IL-10*)	**Forward**	5’-GCTCTTACTGACTGGCATGAG-3’
	**Reverse**	5’-CGCAGCTCTAGGAGCATGTG-3’
Peptidyl arginine deiminase (*PAD4*)	**Forward**	5’-ACAGGTGAAAGCAGCCAGC-3’
	**Reverse**	5’-AGTGATGTAGATCAGGGCTTGG-3’
Interleukin-1β (*IL-1β*)	**Forward**	5’-GAAATGCCACCTTTTGACAGTG-3’
	**Reverse**	5’-TGGATGCTCTCATCAGGACAG-3’
Spleen tyrosine kinase (*Syk*)	**Forward**	5’-GCAGCAGAACAGGCACATTA-3’
	**Reverse**	5’-TCGCTGATCTTGGCATAGTG-3’
Nuclear factor kappa B (*NFκB*)	**Forward**	5’-CTTCCTCAGCCATGGTACCTCT-3’
	**Reverse**	5’-CAAGTCTTCATCAGCATCAAACTG-3’
β-actin	**Forward**	5’-CGGTTCCGATGCCCTGAGGCTCTT-3’
	**Reverse**	5’-CGTCACACTTCATGATGGAATTGA-3’
